# CYRI-B loss promotes enlarged mature focal adhesions and restricts microtubule and ERC1 access to the cell leading edge

**DOI:** 10.1242/jcs.263646

**Published:** 2025-11-24

**Authors:** Jamie A. Whitelaw, Sayantika Ghosh, Sergio Lilla, Savvas Nikolaou, Luke Tweedy, Loic Fort, Nikki R. Paul, Sara Zanivan, Nikolaj Gadegaard, Robert H. Insall, Laura M. Machesky

**Affiliations:** ^1^ CRUK Scotland Institute, Garscube Estate, Switchback Road, Glasgow G61 1BD, UK; ^2^ School of Cancer Sciences, University of Glasgow, Glasgow G61 1QH, UK; ^3^ Department of Biochemistry, University of Cambridge, Cambridge CB2 1GA, UK; ^4^ Division of Biomedical Engineering, School of Engineering, University of Glasgow, Glasgow G12 8LT, UK

**Keywords:** Focal adhesions, Paxillin, Vinculin, Integrins, Bio-ID, ERC1, Actin cytoskeleton, Microtubules, CYRI-B

## Abstract

CYRI proteins promote lamellipodial dynamics by opposing Rac1-mediated activation of the Scar/WAVE complex. This activity also supports resolution of macropinocytic cups, promoting internalisation of surface proteins, including integrins. Here, we show that CYRI-B also promotes focal adhesion maturation and dynamics. Focal adhesions in CYRI-B-depleted cells show accelerated maturation and become excessively large. We probed the composition of these enlarged focal adhesions, using a Bio-ID screen, with paxillin as bait. Our screen revealed changes in adhesion proteins proximal to paxillin suggesting early activation of stress fibre contraction and depletion of the integrin internalisation mediator ERC1. Lack of CYRI-B leads to more stable lamellipodia and accumulation of polymerised actin in stress fibres. This actin acts as a barrier to microtubule targeting for adhesion turnover. Thus, our studies reveal an important connection between lamellipodia dynamics controlled by CYRI-B and microtubule targeting of ERC1 to modulate adhesion maturation and turnover.

## Teaser

**Highlighted Article:** CYRI-B-depleted cells show enlarged stable focal adhesions. A Bio-ID screen revealed a role for CYRI-B in microtubule-mediated adhesion turnover.

## INTRODUCTION

As cells migrate over planar surfaces, they create broad flat membrane protrusions at the front, termed lamellipodia. Activation of the small GTPase Rac1 triggers actin assembly in lamellipodia through binding to the Scar/WAVE complex subunit CYFIP1 (Chen et al., 2010). Binding to Rac1 allows conformational changes of the complex and activation of the Arp2/3 complex to nucleate a branched actin filament network providing the protrusive forces required to extend the plasma membrane (Mullins et al., 1998). The connection of the cell to the surrounding extracellular matrix (ECM) guides migration of individual cells and in multicellular organisms, underpinning fundamental processes such as embryogenesis and cancer metastasis. There have been many different types of cell–ECM adhesions described, such as focal complexes, focal adhesions (FAs), fibrillar adhesions and 3D matrix adhesions (Doyle et al., 2022). However, they all share a common characteristic that the engaged integrins connect to the actin cytoskeleton through a complex of core adhesion proteins (Geiger et al., 2009). Engaged integrins allow the cell to sense and respond to the surrounding environment by converting mechanical stimuli from FAs into biochemical signals, in a process commonly known as mechanotransduction (Humphrey et al., 2014).

FAs form through the engagement of integrins to the matrix along the cell periphery at the lamellipodia tip (Giannone et al., 2007; Zaidel-Bar et al., 2003). Initially adhesions resemble small dot-like structures known as nascent adhesions, which mature and enlarge, changing in protein composition. Over 2000 proteins have been identified as enriched in fibronectin-induced adhesions, but a core of 60 proteins that have been most commonly identified is known as the core adhesome (Horton et al., 2016). Paxillin is one of the earliest proteins recruited to nascent adhesions and is associated with signalling pathways, such as via focal adhesion kinase (FAK, encoded by *PTK2*), through two LD-binding sites located on the N-terminal domain of paxillin (Legerstee and Houtsmuller, 2021; Scheswohl et al., 2008). FAK is responsible for the recruitment of talin proteins to the nascent adhesions which links the cytoplasmic tails of integrins to the actin cytoskeleton (Lawson et al., 2012). This in turn can influence FA size, which links to cell migration speeds (Kim and Wirtz, 2013) and is reported as a measure of integrin signalling during epithelial-mesenchymal transition (EMT) in many cell types (Legerstee and Houtsmuller, 2021; Tsubouchi et al., 2002). Phosphorylation of integrin-mediated adhesion proteins by paxillin and FAK activates the small GTPase Rac1 in a signalling cascade, which in turn activates the Scar/WAVE complex and enhances membrane protrusion (Zaidel-Bar et al., 2005). As the cell moves forward, the nascent adhesions become associated with the lamellipodium–lamellum interface (Alexandrova et al., 2008), where the retrograde flow rate reduces, and adhesions either disappear or enlarge into mature FAs engaged with actin bundles. These recruit additional adaptor and signalling proteins, such as vinculin, zyxin and α-actinin proteins, and begin to exert mechanical forces upon the actin cytoskeleton (Burridge and Guilluy, 2016; deMali et al., 2002). Maturation is a positive feedback loop, triggering further clustering of activated integrins (Humphries et al., 2007) to strengthen the actin–integrin connections, and elongation and strengthening of links with contractile actin stress fibres containing myosin-II (Pellegrin and Mellor, 2007).

As cells migrate, FAs linked to the ECM disassemble, and the disengaged integrins are internalised and degraded or recycled back to the plasma membrane (Moreno-Layseca et al., 2019). This can be facilitated by the protease calpain cleaving integrins and talin proteins (Franco et al., 2004; Kerstein et al., 2017), dynamin and clathrin-mediated endocytosis, and clathrin-independent mechanisms, such as macropinocytosis and caveolin-mediated endocytosis (Maritzen et al., 2015). Membrane trafficking and microtubules play an important dual role in FAs, both in positive trafficking of integrins to nascent adhesions and in trafficking of relaxation or disassembly factors, such as metalloproteases to degrade matrix (Garcin and Straube, 2019; Seetharaman and Etienne-Manneville, 2019; Stehbens et al., 2014). Microtubules are also thought to promote endocytosis at FAs, possibly mediating integrin internalisation (Ezratty et al., 2005). To enhance FA turnover, microtubules are targeted to FA sites by CLASP-mediated capture to the ends of actin stress fibres via a complex of proteins including LL5β (also known as PHLDB2), ERC1 and liprin-α1 (also known as LAR-interacting protein 1 or PPFIA1) (Astro et al., 2014, 2016; Hotta et al., 2010; Lansbergen et al., 2006; Stehbens et al., 2014). These in turn link to talins via the adaptor Kank proteins to release the FA complex of proteins on the cytoplasmic side (Bouchet et al., 2016; Paradzik et al., 2020). ERC1 targeting promotes the internalisation and recycling of surface integrins (LaFlamme et al., 2018) via Rab7-dependent vesicles along microtubules (Astro et al., 2016).

Lamellipodia and adhesion dynamics are fundamental for cell behaviour. We recently showed that loss of the Scar/WAVE complex caused by *NckAP1* deletion had a negative effect on FA turnover and cell migration (Whitelaw et al., 2020). Furthermore, the Scar/WAVE complex has been implicated in the internalisation and recycling of integrins (Rainero et al., 2015). Recently, we identified a novel class of Rac1-interacting proteins that act as negative regulators of the Scar/WAVE complex activation, termed CYFIP-related RAC1 interacting (CYRI) proteins (Fort et al., 2018). There are two isoforms of CYRI proteins in mammals, named CYRI-A and CYRI-B [encoded by the genes *CYRIA*, *CYRIB* (human) and *Cyria*, *Cyrib* (mouse; hereafter denoted *Cyri-a* and *Cyri-b*), formerly known as *FAM49A*, *Fam49a,* and *FAM49B*, *Fam49b*, respectively]. CYRI proteins oppose Rac1-mediated activation of Scar/WAVE and Arp2/3 and thus control cell migration and chemotaxis (Fort et al., 2018), macropinocytic structures (Le et al., 2021; Nikolaou et al., 2024) and pathogen invasion (Yuki et al., 2019). Here, we show that deletion of *Cyrib* (enhances FA assembly during early stages of spreading and alters the recruitment of core FA proteins. FAs become larger and more mature in *Cyri-b*-knockout (KO) cells than controls. We performed a Bio-ID screen to detect changes in composition of FAs in CYRI-B depleted cells. Among the changes, we found that *Cyri-b* KO cells have reduced ERC1 in the vicinity of paxillin by proximity biotinylation and at the leading edge, by immunofluorescence. This paucity of ERC1 is accompanied by reduced microtubule recruitment to the cell periphery, likely promoting the stable enlarged FAs by preventing microtubule-stimulated turnover.

## RESULTS

### FAs are elongated and larger in *Cyri-b* KO cells

**Fig. 1.**
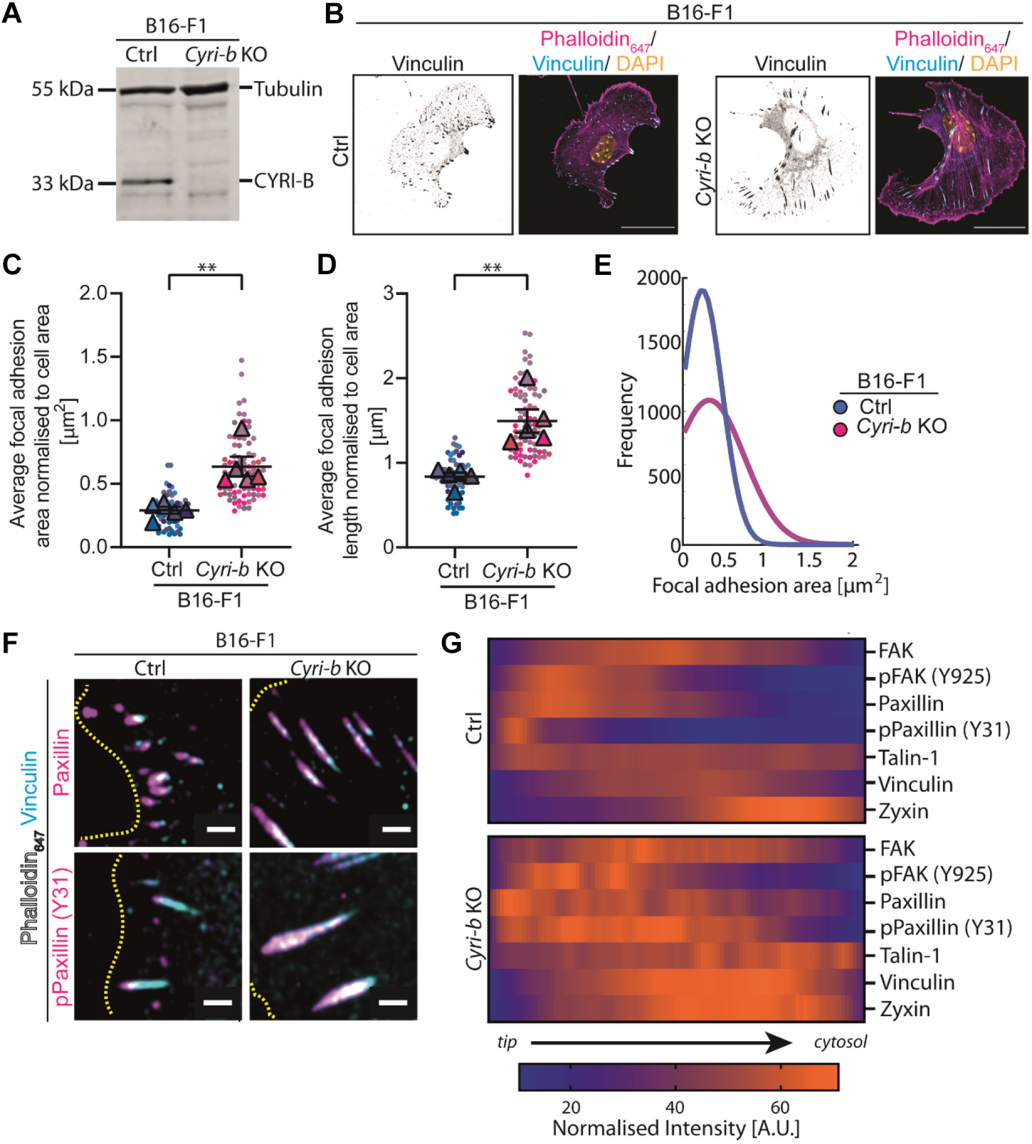
**FAs are elongated and show enhanced phospho-paxillin in *Cyri-b* knockout cells.** (A) Immunoblot of CRISPR-Cas9 knockout of *Cyri-b* in B16-F1 cells. Tubulin as loading control and anti-CYRI-B. Image representative of three independent repeats. (B) FA sizes were compared in B16-F1 Ctrl and *Cyri-b* KO cells. Representative images B16-F1 cells spreading on laminin-coated coverslips and stained with vinculin (cyan), phalloidin (magenta) and DAPI (yellow). Greyscale image of vinculin on the left. Scale bars: 25 µm. (C,D) FA area (C) and FA length (D). A total of 69 control and 79 *Cyri-b* KO cells were analysed from five independent experiments. Results shown in SuperPlot format with error bars representing mean±s.e.m. Analysed with *n*=5 and a paired parametric *t*-test. **P<0.01. (E) An independent analysis of FA area detected by CellProfiler and presented as a line distribution of the frequency. (F,G) Comparisons of FA composition between B16-F1 control and *Cyri-b* KO cells using vinculin antibodies to normalise. (F) Representative images with vinculin (cyan) and the comparative FA antibody (magenta). The leading edge of the cell is highlighted by a dashed yellow line. Scale bars: 2 µm. (G) Profiles of FAs were measured with the intensity normalised to the corresponding vinculin intensity. The colour heat map indicates the average intensity of FA proteins from the FA tip through to the end facing the cytoplasm. Orange represents a high fluorescence intensity (e.g. strong localisation). Purple represents low fluorescence intensity indicating weak localisation within the FA. At least five FAs across five different cells were measured for each antibody. A.U., arbitrary units.

CYRI-B restricts lamellipodia spreading and directed cell migration by dynamically sequestering active Rac1 away from the Scar/WAVE complex (Fort et al., 2018). Nascent adhesions form within the lamellipodia region of migrating cells and coupled with the actin retrograde flow, mature into FAs (Hu et al., 2007). Therefore, we asked how loss of CYRI-B might affect FAs. We deleted *Cyri-b* in B16-F1 mouse melanoma cells using transient CRISPR-Cas9-GFP (Ran et al., 2013). Cas9-GFP-positive B16-F1 cells were sorted by flow cytometry and the clones were tested for the loss of CYRI-B by western blotting (Fig. S1A). As previously reported (Fort et al., 2018), *Cyri-b* KO clones in B16-F1 cells spread to a larger extent than controls (Fig. S1B), as measured by xCelligence and formed large, broad lamellipodia (Fig. 1A,B). For this study, we focused on clone #3 and confirmed the deletion of *Cyri-b* by immunoblotting (Fig. 1A; Fig. S1A).

Loss of CYRI-B resulted in large, elongated FAs spread throughout the lamellipodium and cell body of B16-F1 cells (Fig. 1B–E). Quantification of FA area using CellProfiler showed that the *Cyri-b* KO cells had an increased frequency of larger FAs (Fig. 1E). We confirmed the enlargement of FAs in *Cyri-b^fl/fl^* mouse embryonic fibroblasts (MEFs) with Cre-ERT2 (Fort et al., 2018), which deletes *Cyri-b* upon addition of 4-hydroxytamoxifen. MEFs generally displayed larger FAs than B16 F1 cells, but these were further enlarged upon deletion of *Cyri-b* (Fig. S1C,D).

To explore maturation status of the larger FAs in CYRI-B depleted cells, we probed the distribution of key protein components of the adhesion machinery. By creating a heat map of the intensities of each protein and averaging this over several FAs (Fig. 1F), measuring from the most peripheral point (tip) towards the cell centre (cytosol) (Fig. S1E), we compared the distributions of FAK, paxillin, talin-1 and zyxin to that of vinculin (Fig. 1F,G; Fig. S1F,G). Paxillin displayed a similar profile in the control (Ctrl) and *Cyri-b* KO cells but showed a broader distribution in the *Cyri-b* KO cells. There was also a large increase in the intensity and breadth of paxillin phosphorylated at Y32 [pPaxillin (Y31)], which has been shown to be important for cell migration (Petit et al., 2000) (Fig. 1G; Fig. S1F). The distribution of FAK was similar between Ctrl and *Cyri-b* KO cells (Fig. 1G; Fig. S1F). We also checked the phosphorylation of FAK at Y925 [pFAK (Y925)] owing to its role in cell migration through its activation of the p130Cas/Rac1 signalling pathways (Deramaudt et al., 2011). However, similar to FAK, pFAK (Y925) showed only slight changes in distribution in the *Cyri-b* KO cells (Fig. 1G; Fig. S1F).

Talins directly connect to both integrins and F-actin (Das et al., 2014; Jin et al., 2015), whereas vinculin is recruited to talins and reinforces the F-actin anchoring (Bays and DeMali, 2017; Boujemaa-Paterski et al., 2020). As expected, vinculin and talin localisation spanned nearly the whole FA in both the Ctrl and *Cyri-b* KO cells (Fig. S1F). However, of note, talin-1 exhibits prominent intensity peaks to the rear half of the FA in the *Cyri-b* KO cells that are not observed in the control cells (Fig. 1G; Fig. S1F). Furthermore, where vinculin is spread in the FAs similarly in Ctrl and *Cyri-b* KO cells, the intensity of vinculin is greater in the *Cyri-b* KO cells and zyxin is similar but has a broader distribution in the *Cyri-b* KO cells (Fig. 1G; Fig. S1F). In summary, FAs in CYRI-B depleted cells show enhanced phospho-paxillin and enhanced recruitment of vinculin and talin-1, suggesting that the larger FAs are more mature, which might reflect reduced turnover dynamics.

**Fig. 2.**
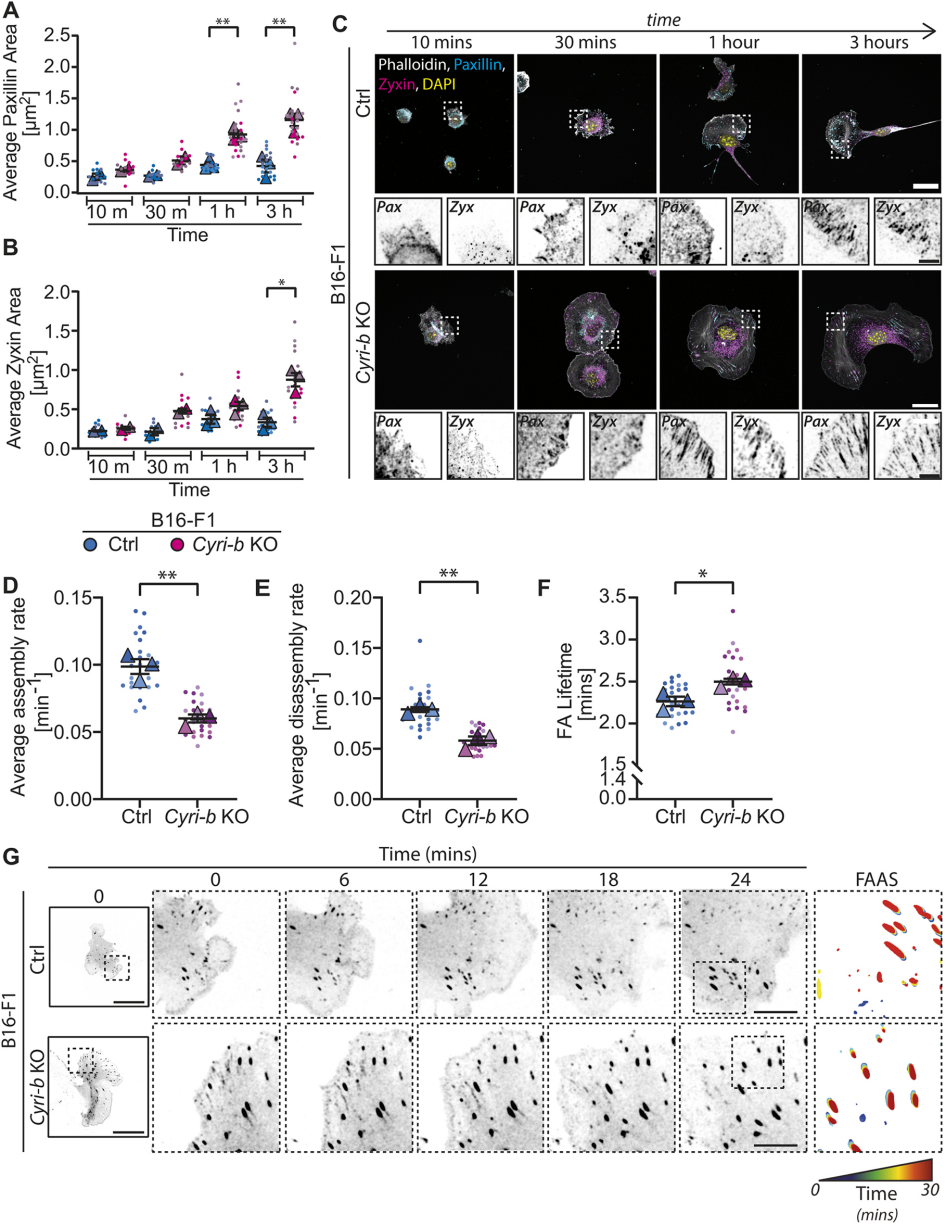
**Adhesion dynamics are altered in the *Cyri-b* KO cells.** (A–C) The formation and maturation of FAs in B16-F1 cells from initial seeding to cells spreading. Phalloidin (white) was used as a marker for the cell size, paxillin (cyan) was used as an early FA marker and zyxin (magenta) was used as a later marker for mature FAs. Cells were trypsinised and seeded for the indicated time before fixation. (A) The average paxillin area and (B) the average zyxin area over time for the control and *Cyri-b* KO cells. 15 cells from 10–30 min and 25 cells for 1–3 h analysed from ≥2 independent experiments. Results shown in SuperPlot format with error bars representing mean±s.e.m. Two-tailed paired *t*-test comparing control and *Cyri-b* KO cells on *n*=2 (10 and 30 min) or *n*=3 (1 and 3 h) experiments. *P<0.05, **P<0.01. (C) Representative images for the time course experiment. Scale bars: 25 μm (main images); 2.5 μm (magnifications). (D–G) FA dynamics of 27 cells from three independent experiments. Cells expressing pEGFP-Paxillin were assessed for their FA assembly rates (D) and disassembly rates (E). (F) The lifetimes of the FAs. Results shown in SuperPlot format with error bars representing mean±s.e.m. Statistical differences determined by a two-tailed paired *t*-test comparing control and *Cyri-b* KO cells. *P<0.05, **P<0.01. (G) Representative images of FA turnover over the 30-min time course. For the FAAS, there adhesions are colour coded through time from blue at the start to red at the end of the experiment. Scale bar: 25 μm (overview image); 5 μm (time magnifications).

We next examined how the larger FAs in *Cyri-b* KO cells formed and matured over time. B16-F1 cells were replated and fixed at different time points during adhesion to allow observation of a time progression from early focal complex formation to more mature FAs (Geiger et al., 2009). We used paxillin as a marker of early focal complex formation, which we expected to remain through to FA maturity, and also zyxin as a marker for mature FAs (Legerstee and Houtsmuller, 2021). *Cyri-b* KO cells recruited proteins, such as paxillin, to the focal complex as early as 30 min where the adhesion sizes were larger over the 3-h time-course (Fig. 2A–C). Similarly, zyxin was also observed in the FAs after 30 min (Fig. 2A–C), indicating that either these early focal complexes displayed markers of mature FAs or that these early focal complexes matured rapidly to FAs (Fig. 2B,C). We next investigated the dynamics of the large FAs in the *Cyri-b* KO compared to the control B16-F1 cells by measuring the assembly and disassembly rates, and the lifetime of the adhesions after the cells had been allowed to attach and migrate in a steady state. Live imaging of the cells expressing pEGFP-Paxillin were captured over a 30-min time course and analysed using the FA analysis server (FAAS; Berginski and Gomez, 2013). Here, we observed that the adhesions in the control cells were able to form and disassemble much faster than those in the *Cyri-b* KO cells (Fig. 2D,E,G; Movie 1). It was apparent when calculating the average longevity of the adhesions that, on average, the FAs in the *Cyri-b* KO persisted for longer (Fig. 2F). Overall, this indicates that these large FAs in the *Cyri-b* KO are more stable than those of the control cells.

### The large FAs in *Cyri-b* KO cells are not solely due to increased Rac1 activity

**Fig. 3.**
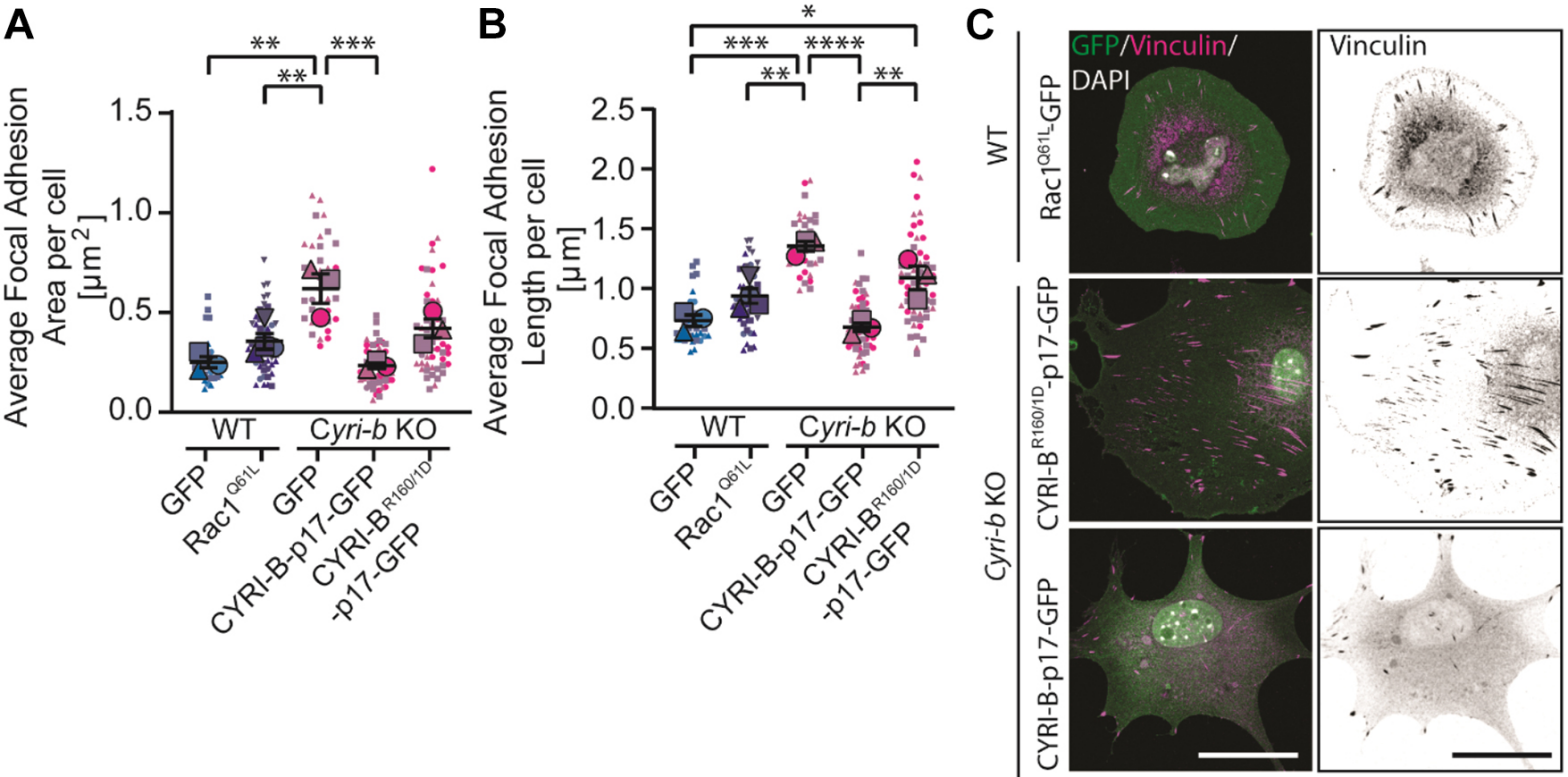
**Increased Rac1 activity alone does not account for the enlarged FAs in *Cyri-b* KO cells.** (A–C) FA sizes in B16-F1 cells expressing different GFP constructs to assess whether increased Rac1 is activity is responsible for the large FAs in the *Cyri-b* KO cells. B16-F1 WT cells expressing pEGFP-Rac1^Q61L^ or *Cyri-b* KO cells rescued with CYRI-B-p17–GFP or CYRI-B^R160/1D^-p17–GFP (Rac1-binding mutant). (A) FA area. (B) FA length. *n*=35 WT+GFP only, 53 WT+Rac1^Q61L^-GFP, 35 *Cyri-b* KO, 56 *Cyri-b* KO+CYRI-B-p17-GFP and 57 *Cyri-b* KO+CYRI-BR160/1D-p17-GFP cells analysed from three independent experiments, shown by the different symbols. Results shown in SuperPlot format with error bars representing mean±s.e.m. for *n*=3 independent experiments. Statistical differences determined by one-way ANOVA with Tukey's post hoc test. *P<0.05, **P<0.01, ***P<0.001, ****P<0.0001. (C) Representative images of FA in cells expressing GFP fusion constructs. Left-hand side shows merge with GFP (green), vinculin (magenta) and DAPI (white). Right side shows images of vinculin in greyscale. Scale bars: 25 μm.

During spreading, α5β1 integrin signalling leads to Rac1 activation at the leading cell edge and subsequent lamellipodia protrusions (Price et al., 1998). This increases FAK and paxillin phosphorylation, leading to increased activation of the p130Cas/Dock180/Rac1 pathways in a positive feedback loop (Valles et al., 2004). As growth of the adhesions progresses, Rac1 is replaced by RhoA, activating contractile forces along the FAs (Arthur and Burridge, 2001). Loss of CYRI-B causes the cells to form large lamellipodia due to an increased activity of active Rac1, inducing Scar/WAVE complex activity (Fort et al., 2018). We speculated that increased Rac1 activity in the *Cyri-b* KO could be modifying the FA turnover. To test this, we expressed constitutively active mutant Rac1^Q61L^–GFP into B16-F1 wild-type (WT) cells. FA sizes were significantly larger in Rac1^Q61L^–GFP expressing cells, but importantly these FAs were still significantly smaller than those of *Cyri-b* KO cells (Fig. 3A–C). We also rescued the *Cyri-b* KO cells with CYRI-B-p17–GFP an internally tagged CYRI construct in which GFP was inserted after residue 17 of CYRI-B (Le et al., 2021). CYRI-B-p17–GFP rescue restored normal FA sizes. We rescued with CYRI-B^R160/161D^-p17–GFP, a construct with mutations preventing Rac1 interaction (Fort et al., 2018), which conferred a reduction of FA sizes but only to a level similar to cells expressing Rac1^Q61L^ (Fig. 3A–C). Overall, this suggests that increased Rac1 activity in the *Cyri-b* KO cells only partially contributes to the large FA size.

### A BioID screen for paxillin interactions reveals altered FA networks in *Cyri-b* KO cells

**Fig. 4.**
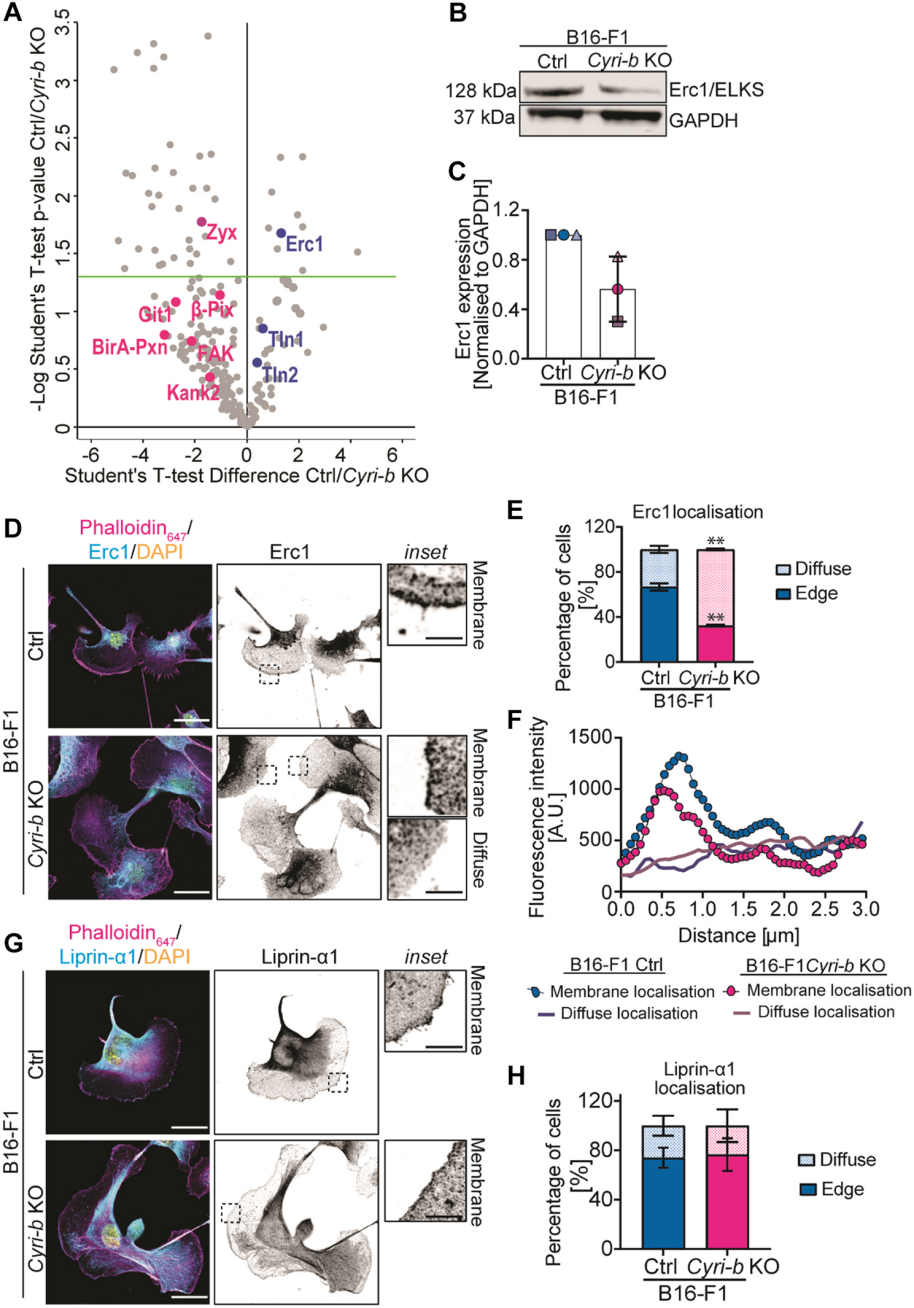
**BioID screen of paxillin reveals decreased association with ERC1 in *Cyri-b* KO cells.** (A) Volcano plot displaying the results from the proximity biotinylation screen of paxillin in B16-F1 control and *Cyri-b* KO cells. Proteins enriched in proximity to paxillin in *Cyri-b* KO cells are shown in magenta and proteins enriched in control cells shown in blue. Proteins above the green horizontal line are enriched in either control or *Cyri-b* KO cells. See also Fig. S2 and Table S1 for details of other enriched proteins. *P*<0.05. (B) Representative western blot of endogenous ERC1 levels in B16-F1 control and *Cyri-b* KO cells. (C) Quantification of ERC1 from western blotting normalised to GAPDH loading control. Error bars represent mean±s.d. from three independent experiments. (D) Representative images of ERC1 localisation using an anti-ERC1 antibody. Actin cytoskeleton (magenta), ERC1 (cyan) and DAPI (yellow). Magnifications on the right depict ERC1 localisation either at the membrane or as a diffuse cytosolic staining. Scale bars: 25 μm (main images); 5 μm (magnifications). (E) Quantification of ERC1 localisation to cell edge (solid colour) or diffuse in the cytoplasm (coloured dots). *n*=61 control and 65 *Cyri-b* KO cells analysed from three independent experiments and converted to percentages. Mean±s.d. Statistical differences determined by two-tailed unpaired *t*-test with Welch's correction. **P<0.01. (F) FIJI plot profile fluorescence intensity of the localisation of ERC1 staining. The lines with circles represent the average intensity of the ERC1 signal from cells with a membrane localisation. The lines without circle points represents the intensity of diffuse staining showing a lack of intensity at the membrane. The distance measured was 3 μm from the leading edge into the cell. *n*=19 control and 19 *Cyri-b* KO cells. A.U., arbitrary units. (G) Representative images of liprin-α1 localisation using an anti-liprin-α1 antibody (cyan), phalloidin (actin cytoskeleton; magenta) and DAPI (yellow). The liprin-α1 channel is displayed in greyscale to the right-hand side. Scale bars: 25 μm (main images); 5 μm (magnifications). Magnifications depict liprin-α1 localisation at the membrane. (H) Quantification of liprin-α1 at the plasma membrane (solid colour) versus diffuse cytoplasmic staining (coloured dots). *n*=42 control and 54 *Cyri-b* KO cell analysed from three independent experiments and converted to percentages. Mean±s.d. Statistical differences determined by unpaired two-tailed *t*-test with Welch's correction with no significance reached.

To identify additional factors that might affect FA maturation dependent on CYRI-B, we used paxillin as the bait in a proximity biotinylation Bio-ID experiment (Dong et al., 2016) (Fig. S2A–C). Proximity biotinylation of paxillin was previously used to provide insight into the molecular composition of FAs (Chastney et al., 2020; Dong et al., 2016). Indeed, our Bio-ID screen identified well-known FA proteins such as talin-1, -2, FAK, adhesion regulators, such as Kank2, small GTPase interactors, such as GIT1 and β-PIX (also known as ARHGEF7) and actin-binding proteins, such as Shroom2 and Shroom4 in the larger FAs of *Cyri-b* KO cells (Fig. 4A; Fig. S2D,E, Table S1), although several of these were below our cutoff for significance for *P*-value (Fig. 4A, green line). Interestingly, zyxin, a protein found in more mature FAs, was significantly enriched in the *Cyri-b* KO adhesions compared to the control cells, in agreement with the FAs in the *Cyri-b* KO cells being more mature and with our immunofluorescence analysis (Figs 1G, 2B). On the other hand, the cytoskeleton and membrane trafficking adaptor protein, Rab6-interacting protein (ERC1) was significantly depleted in the proximity of adhesions of *Cyri-b* KO cells (Fig. 4A). ERC1 mediates displacement of cytoplasmic adhesion complex proteins, thus promoting the internalisation of surface integrins via clathrin-mediated and clathrin-independent endocytosis (Astro et al., 2016; Pellinen et al., 2006).

### ERC1 but not liprin-α1 is affected by the loss of CYRI-B

Owing to its importance in integrin internalisation, we investigated ERC1 depletion at *Cyri-b* KO FA further. Immunoblotting showed that that ERC1 total protein levels are reduced in *Cyri-b* KO B16-F1 cells (Fig. 4B,C). Moreover, ERC1 is thought to form a complex with liprin-α1 and LL5β and localise to the leading edge of migrating cells (Astro et al., 2014; Hotta et al., 2010). ERC1 has a clear localisation to the leading edge in ∼70% of control cells but this was reduced to ∼30% in *Cyri-b* KO cells (Fig. 4D,E). Moreover, localisation of ERC1 at the leading edge of *Cyri-b* KO cells formed a tighter intensity peak, with a reduced overall fluorescence intensity (Fig. 4F). Conversely, liprin-α1, the complex partner of ERC1, which marks synaptic vesicle docking sites in neuronal cells (Astro et al., 2016; Ko et al., 2003; Liang et al., 2021), localised to the leading edge in ∼70% of both the control and *Cyri-b* KO cells (Fig. 4G,H), suggesting that ERC1 depletion is relatively specific following the loss of CYRI-B and is in line with a previous study showing that liprin-α1 localisation does not depend on ERC1 (Astro et al., 2016). To ask whether ERC1 interacted with CYRI-B directly, we performed a GFP-trap experiment with GFP–CYRI-B and probed for endogenous ERC1; however we did not detect any interaction (Fig. S2F). This suggests that the effect of CYRI-B depletion on ERC1 localisation is likely to be indirect.

**Fig. 5.**
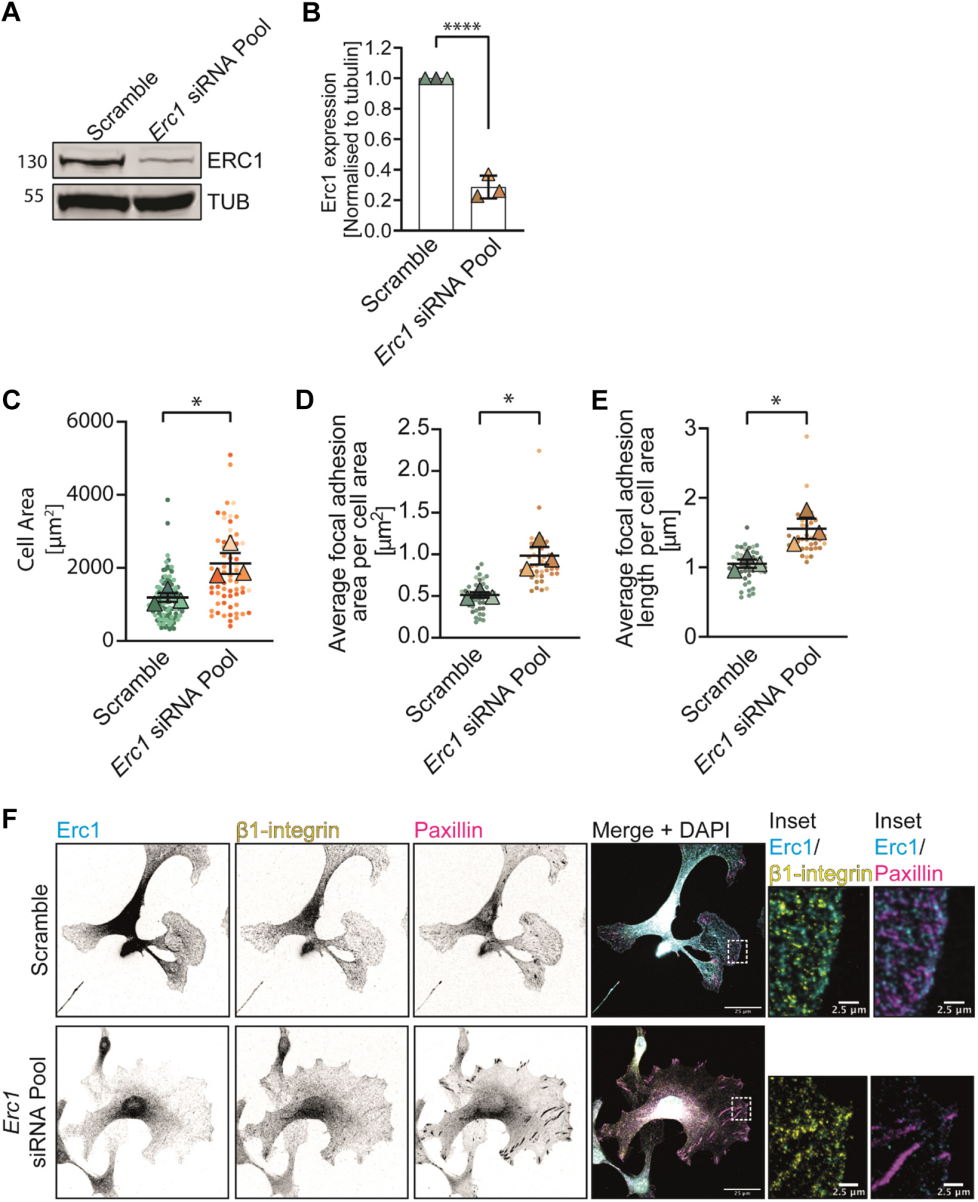
**Depletion of ERC1 increases FA sizes.** Downregulation of *Erc1* in B16-F1 cells using 10 nM specific siRNAs pooled. (A) Representative western blot of ERC1 levels in either B16-F1 cells treated with a scramble or pooled siRNA against Erc1. Tubulin (TUB) was used as loading control. (B) Western blot quantification of ERC1 levels in B16-F1 scramble or ERC1 siRNA pool from three independent experiments. Mean±s.d. Statistical differences determined by two-tailed unpaired *t*-test. ****P<0.0001. (C) Cell area of B16-F1 scramble or *Erc1* siRNA pool. 106 scramble and 63 cells analysed from three independent experiments. Results shown in SuperPlot format with error bars representing mean±s.e.m. Statistical differences determined by two-tailed paired *t*-test on the independent averages from *n*=3 experiments. *P<0.05. (D,E) Average FA area per cell (D) and average FA length (E). 42 scramble and 42 ERC1 knockdown cells analysed from three independent experiments. Results shown in SuperPlot format with error bars representing mean±s.e.m. Two-tailed paired *t*-test on the independent average from *n*=3 experiments. *P<0.05. (F) Representative images for data in C–E. ERC1 (cyan), active β1-integrin (yellow) or paxillin (magenta). Scale bars: 25 μm (main image); 2.5 μm (magnified views on the right).

We reasoned that if loss of *Cyri-b* affects FA size via a reduced association of ERC1 with FAs, then depletion of ERC1 should enhance FA size. Using a pool of small interfering RNAs (siRNAs) specific to *Erc1*, we achieved a greater than 70% reduction in ERC1 protein levels (Fig. 5A,B). B16-F1 cells depleted of ERC1 resembled *Cyri-b* KO cells (Fig. 1C), displaying a larger cell area (Fig. 5C) and large elongated FAs (Fig. 5D–F). This supports our hypothesis that loss of *Cyri-b* affects adhesions and spreading at least partly via interfering with ERC1 recruitment to FAs, which in turn affects FA dynamic turnover.

### Loss of *Cyri-b* or ERC1 similarly impairs integrin internalisation

**Fig. 6.**
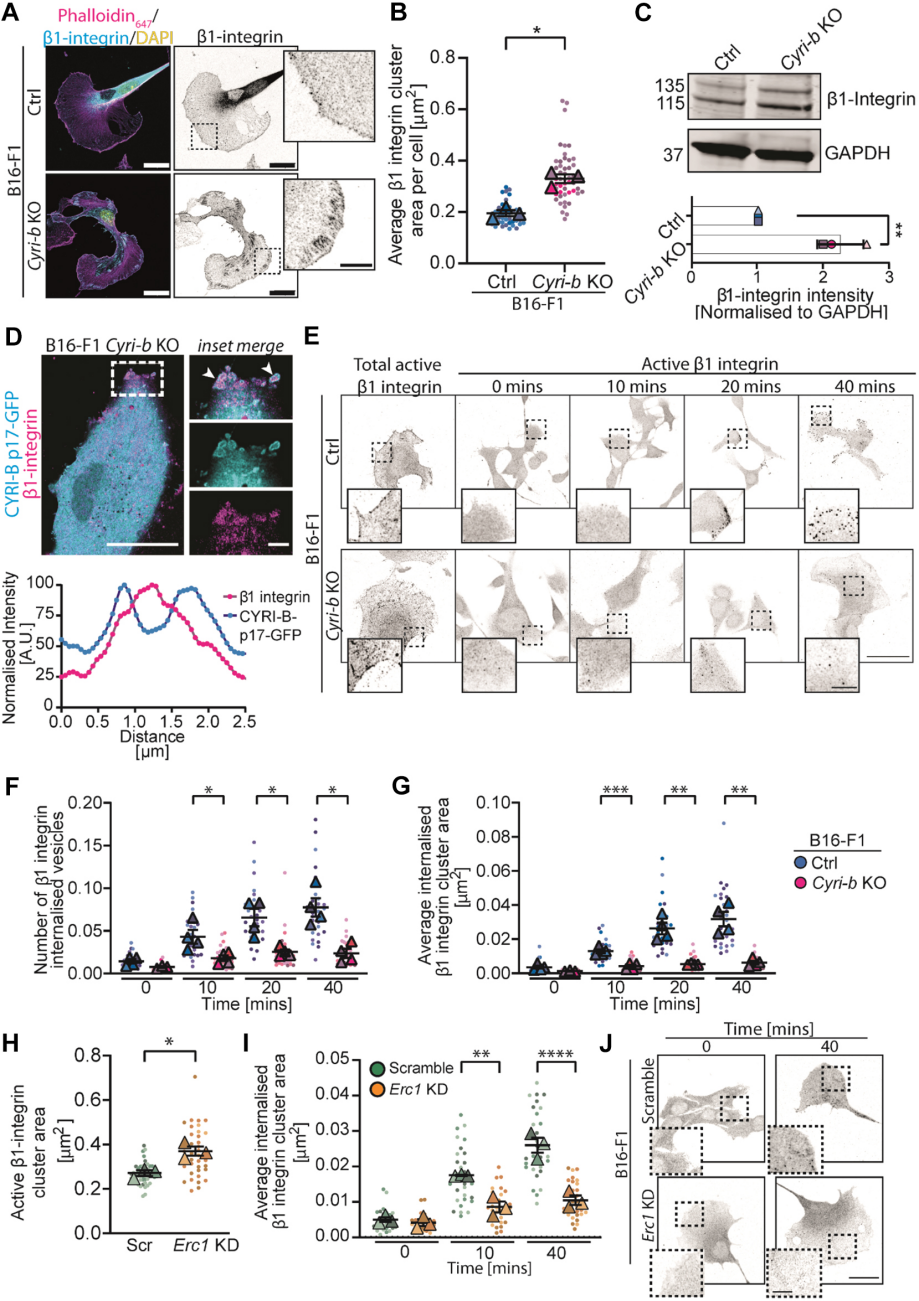
**Loss of *Cyri-b* or *ERC1* reduces integrin internalisation.** (A) Immunofluorescence images of active β1-integrin staining (cyan), actin cytoskeleton (magenta) and DAPI (yellow). Right-hand image: active β1-integrin staining in greyscale. Scale bars: 20 μm (main images); 5 μm (magnified views on the right). (B) Quantification of the average active β1-integrin cluster (integrin-β1-positive adhesion) area in B16-F1 control and *Cyri-b* KO cells. 45 control and 50 *Cyri-b* KO cells analysed from three independent experiments. Results shown in SuperPlot format with error bars representing mean±s.e.m. Two-tailed paired *t*-test on *n*=3 experiments. *P<0.05. (C) Western blot and quantification of total β1-integrin levels in control and *Cyri-b* KO cells from three independent experiments. GAPDH was used as loading control. Mean±s.e.m. Two-tailed unpaired *t*-test. **P<0.01. (D) Live imaging of B16-F1 *Cyri-b* KO cells rescued with CYRI-B-p17-GFP (cyan) and β1-integrin-mCherry (magenta). White arrowheads in magnified view of boxed area highlight β1-integrin-positive structures surrounded by CYRI-B. Scale bars: 25 μm (main image); 5 μm (magnified views on the right). Intensity profile of these β1-integrin-containing structures shows two peaks of CYRI-B signal intensity (cyan) around the peak of β1-integrin intensity (magenta) from 48 different vesicles, from three independent experiments. A.U., arbitrary units. (E–G) Active β1-integrin internalisation comparison between B16-F1 control and *Cyri-b* KO cells. (E) Representative images of internalised active β1-integrin. Total active β1-integrin characterises the normal β1-integrin localisation within the cells prior to the assay. Time course of active β1-integrin internalisation before an acid wash to remove any extracellular bound antibody. Scale bars: 20 μm (main images); 5 μm (magnified views underneath). (F,G) Number of active β1-integrin internalised vesicles (F) and average internalised active β1-integrin cluster area normalised to cell area over time normalised to cell area (G). Results shown in SuperPlot format with error bars representing mean±s.e.m. *n*=30 cells for each condition analysed from four independent experiments. One-way ANOVA with Sidak's multiple comparison post test on *n*=4 independent experiments. *P<0.05, **P<0.01, ***P<0.001. (H,I) Active β1-integrin cluster area between the scramble control and *Erc1* siRNA KD. 42 scramble and 42 *Erc1* knockdown cells analysed from three independent experiments (H) and average active β1-integrin internalised between scramble control and *Erc1* siRNA KD. 30 scramble and 30 *Erc1* knockdown cells analysed from three independent experiments (I). Results shown in SuperPlot format with error bars representing mean±s.e.m. Two-tailed paired *t*-test on the independent average from *n*=3 experiments. *P<0.05, **P<0.01, ****P<0.0001. (J) Representative images of results in H and I for internalised active β1-integrin internalisation in B16-F1 scramble or *Erc1* KD cells. Times shown are 0 and 40 min. Scale bars: 25 μm main images); 5 μm (magnified views underneath).

Depletion of ERC1 has been previously linked to a reduction of internalised β1-integrin receptors and reduced lamellipodial persistence and migration (Astro et al., 2014). We hypothesised that the reduced ERC1 expression in the *Cyri-b* KO cells might increase β1-integrin display at the cell surface. Indeed, we detected an increase in β1-integrin FA area on the surface of migrating *Cyri-b* KO B16-F1 cells (Fig. 6A,B) that was comparable to what we observed for other FA markers (Fig. 1C). We also observed a twofold increase in total β1-integrin levels in *Cyri-b* KO cells (Fig. 6C).

Recent work from our laboratory has demonstrated that CYRI-A and CYRI-B are involved in macropinocytosis leading to the bulk internalisation of integrins (Le et al., 2021). Here, using B16-F1 *Cyri-b* KO cells, rescued with CYRI-B-p17–GFP and β1-integrin–mCherry we performed super-resolution live imaging and observed β1-integrin being internalised on vesicular structures surrounded by CYRI-B (Fig. 6D; Movie 2) similar to what was previously reported in other cell types (Le et al., 2021).

We next asked whether β1-integrin internalisation was affected in *Cyri-b* KO cells. Active β1-integrin antibodies were allowed to bind to the integrin extracellular domain and then to internalise for an allocated time before being removed from the extracellular surface. We observed a steady increase in the number of internalised vesicles containing active β1-integrin in the control cells (Fig. 6E,F), which also resulted in a larger internal pools of vesicles containing active β1-integrin (Fig. 6G). In contrast, the *Cyri-b* KO cells had significantly fewer and smaller active β1-integrin-containing vesicles internalised (Fig. 6E,F). Overall, we find a defect in active β1-integrin internalisation in the *Cyri-b* KO B16-F1 cells resulting in an increase in active β1-integrin on the cell surface and in agreement with Le et al. (2021).

ERC1 is important for the internalisation of active integrins from the leading edge of migrating cells (Astro et al., 2014, 2016). Similar to the *Cyri-b* KO cells, the *Erc1* knockdown (KD) cells had more active β1-integrin present at the surface (Fig. 6H) and were much slower to internalise this into the cells (Fig. 6I,J). This confirms previous data that ERC1 promotes active integrin internalisation (Astro et al., 2014, 2016) and supports our hypothesis that depletion of ERC1 from the leading edge of *Cyri-b* KO cells contributes to the enlarged FA phenotype.

### *Cyri-b* loss might impede ERC1 access to FA sites due to enhanced actin and myosin engagement with the cell periphery

**Fig. 7.**
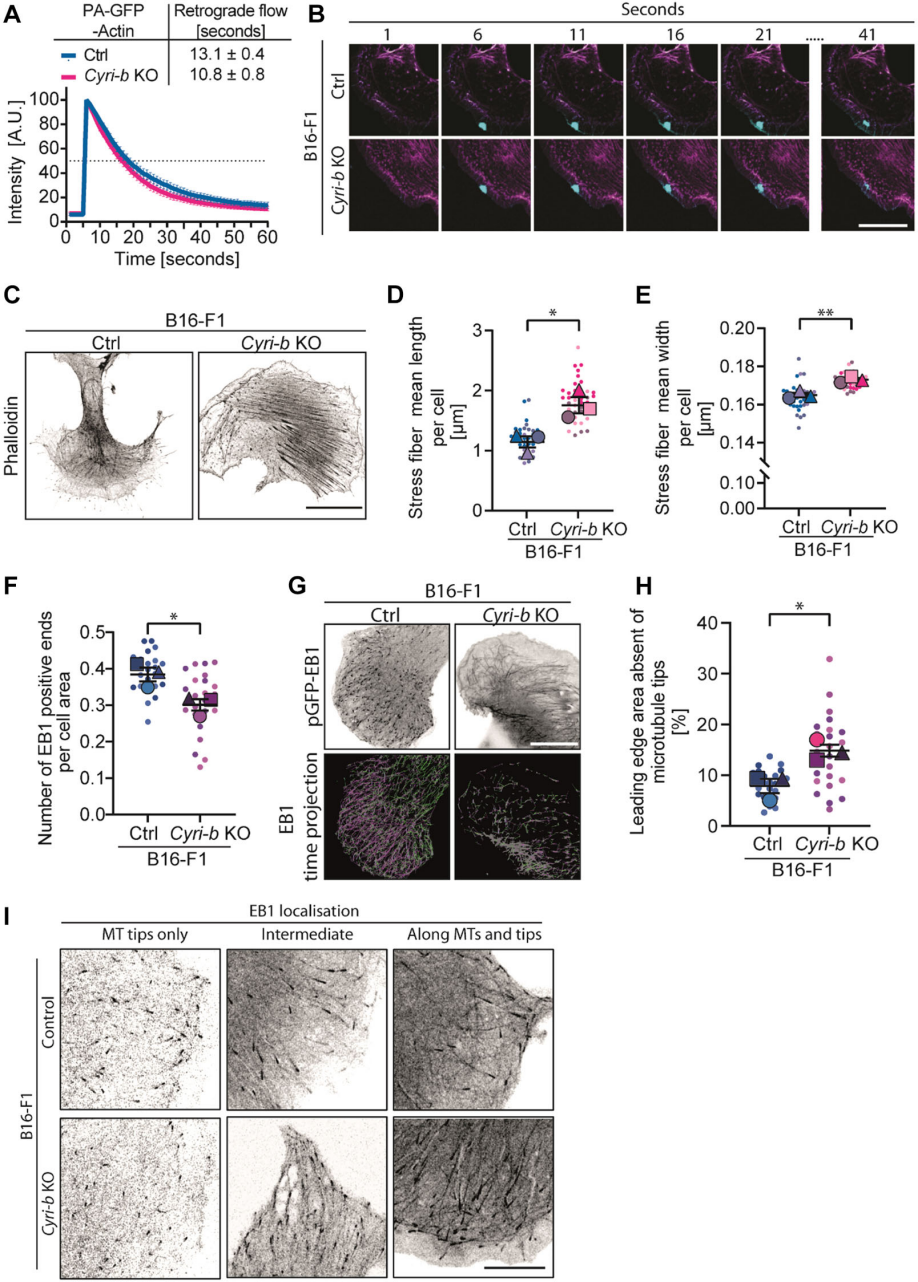
**ERC1 trafficking is affected by reduced microtubule ends, which depend on normal actin dynamics and contractility.** (A,B) The actin retrograde flow was assessed in B16-F1 control and *Cyri-b* KO cells. (A) The half-time from activating PA-GFP actin at the lamellipodia edge to flow into the lamella region of the cell. The peak in intensity correlates with photoactivation after 5 s. Intensity plot over time from 60 cells from three independent experiments where the error bars represent mean±95% c.i. Average retrograde flow time is shown in the upper box (mean±s.d.) (B) Representative images of photoactivation of PA-GFP–Actin (cyan) and the actin cytoskeleton shown using LifeAct-TagRed (magenta) at various timepoints. Scale bar: 20 μm. (C) Representative images of stress fibres quantified using phalloidin staining to highlight the F-actin cytoskeleton. Scale bar: 25 μm. (D,E) Average stress fibre length (D) and average stress fibre thickness (E). 40 cells measured from three independent experiments. Error bars represent mean±s.e.m. Statistical significance determined using an unpaired two-tailed *t*-test. *P<0.05, **P<0.01. (F) The number of EB1-positive microtubule ends normalised to cell area. 25 cells measured from three independent experiments. Results shown in SuperPlot format with error bars representing mean±s.e.m. Statistical significance determined using an unpaired two-tailed *t*-test. *P<0.05. (G) Representative images of *Cyri-b* KO B16-F1 cells expressing GFP–EB1 in greyscale (top) and a time projection (bottom) where magenta shows EB1 travel towards the leading edge and green as the EB1 travelling to the cytoplasmic region. Scale bar: 25 μm. (H) Quantification of the area at the leading edge without microtubules as a percentage of the cell area. 25 cells measured from three independent experiments. Results shown in SuperPlot format with error bars representing mean±s.e.m. Statistical significance determined using an unpaired two-tailed *t*-test. *P<0.05. (I) Representative images of GFP–EB1 localisation in B16-F1 control and *Cyri-b* KO cells as quantified in H. Scale bar: 10 µm.

We further explored possible mechanisms by which CYRI-B depletion might enhance FAs and prevent ERC1 reaching the leading edge. As FAs form through the activation of integrins and mature under the influence of actin retrograde flow, we speculated that actin retrograde flow might be different in *Cyri-b* KO cells, disrupting normal adhesion maturation. As the *Cyri-b* KO cells form broad lamellipodia and have more active-Rac1 (Fort et al., 2018), we measured the actin retrograde flow in B16-F1 cells. Actin was marked in the lamellipodia tip by photoactivatable-GFP–actin (PA-GFP–Actin) and over time we observed that there was no significant difference in the actin retrograde flow between control and *Cyri-b* KO cells (Fig. 7A,B; Movie 3). Therefore, we conclude that the enlarged FAs in the *Cyri-b* KO cells are not likely caused by changes in actin retrograde flow.

**Fig. 8.**
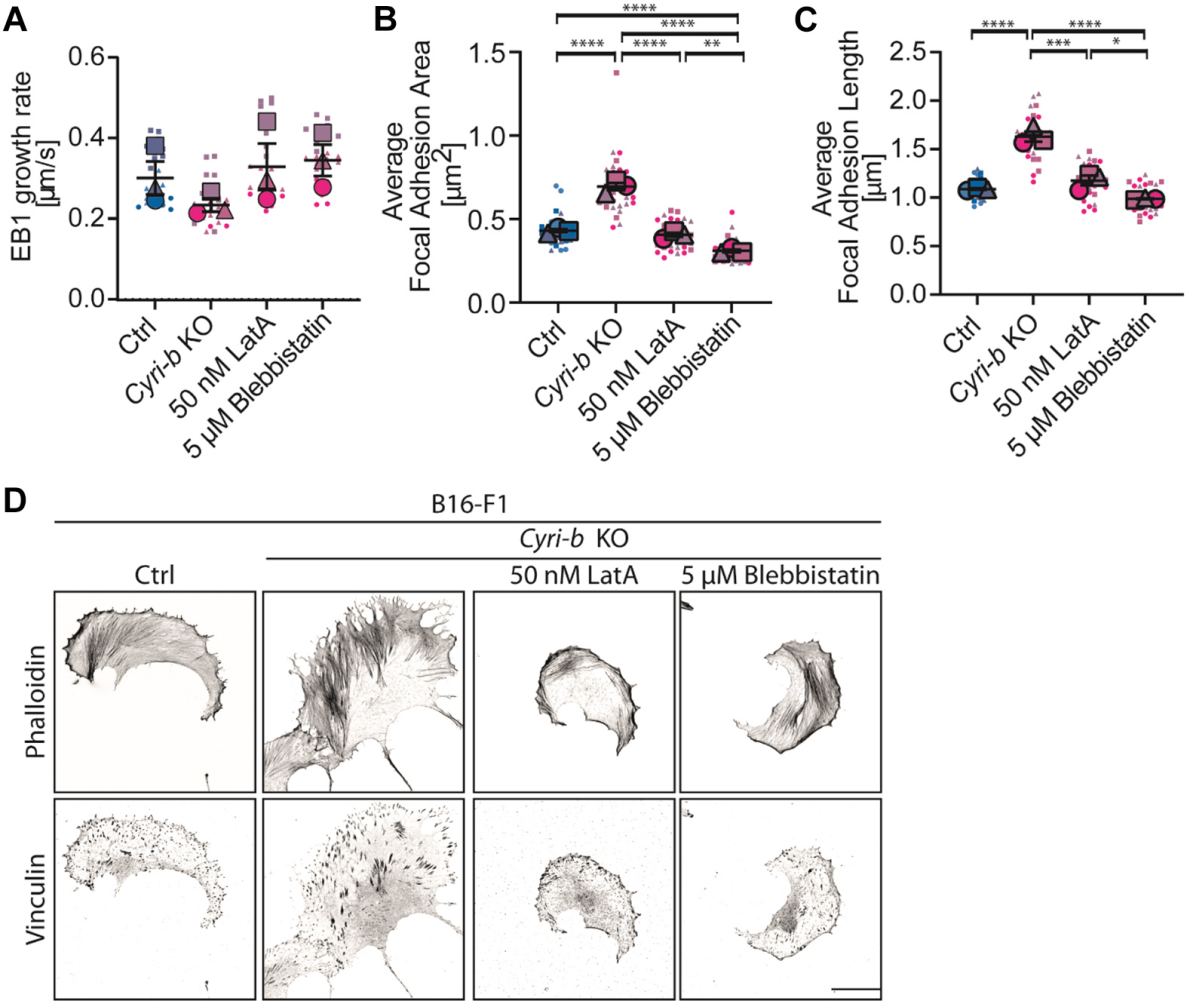
**Loosening the actin tension and contractility restores normal microtubule growth rates and FA sizes.** (A) EB1 growth rates in B16-F1 control and *Cyri-b* KO cells with inhibitors. 25 cells were analysed over three independent experiments. Results shown in SuperPlot format with error bars representing mean±s.e.m. Statistical significance measured by a one-way ANOVA with Tukey's multiple comparison test; significance was not reached. (B–D) Low dose chemical disruption to the actin cytoskeleton or cell contractility with 50 nM LatA or 5 µM blebbistatin, respectively. (B,C) FA area (B) and FA length (C) in B16-F1 control or *Cyri-b* KO cells with inhibitors. 30 cells were analysed over three independent experiments. Results shown in SuperPlot format with error bars representing mean±s.e.m. Statistical significance measured by a one-way ANOVA with Tukey's multiple comparison test. *P<0.05, **P<0.01, ***P<0.001, ****P<0.0001. (D) Representative images of FA sizes in B16-F1 control and *Cyri-b* KO cells treated with 50 nM LatA or 5 µM blebbistatin as quantified in B,C. Scale bar: 25 μm.

We noticed an increase in F-actin cables throughout the *Cyri-b* KO cells. This was not surprising, as mature FAs connect with actin stress fibres and regulate tension via zyxin and α-actinin (Burridge and Guilluy, 2016). Quantitative image analysis revealed that the *Cyri-b* KO cells had longer and thicker actin stress fibres when compared to the control cells (Fig. 7C–E). Next, we asked whether the reduction of microtubule growth rates could be due to the contractile tension or steric hindrance from the strong actin stress fibres and/or a blockage from excessive actin accumulation at the leading edge of the cell. To answer this, we used either a low dose treatment of latrunculin A (LatA) to reduce actin assembly at the leading edge (Yarmola et al., 2000) or we treated the cells with a low dose of blebbistatin to inhibit myosin-II contractility (Martino et al., 2018). Both low dose LatA and blebbistatin treatment rescued the end-binding-1 (EB1; also known as MAPRE1) growth rates in the *Cyri-b* KO cells to that of control cells (Fig. 8A; Movie 4). Furthermore, these treatments also rescued FA sizes in the *Cyri-b* KO cells (Fig. 8B–D).

Next, we looked at microtubule dynamics to see whether microtubule positive end tracking was altered. The arrival of ERC1 is thought to displace the complex of FA proteins and allow the internalisation and recycling of integrins from the surface (Astro et al., 2016; Bouchet et al., 2016; Paradzik et al., 2020). Here, we used GFP-tagged EB1 to track the growth rates of microtubules. We observed a drastic reduction in the number of EB1-positive ends in the *Cyri-b* KO cells (Fig. 7F). Furthermore, by tracking EB1 movement at the tips, we determined that the microtubules in the *Cyri-b* KO cells did not reach the lamellipodia edge. This led to the *Cyri-b* KO cells having a larger area at their leading edges that was devoid of microtubules (Fig. 7G,H). We also observed an abnormal EB1 localisation in ∼50% of the *Cyri-b* KO cells covering the whole microtubule lattice. In comparison, this was only observed in the control cells 10% of the time (Fig. 7I). We queried the potential functional consequences of this, using a previously described nocodazole washout experiment, whereby nocodazole destabilisation of microtubules prevents microtubule ends from reaching FA and destabilising them (Kaverina et al., 1999). Subsequent washout of nocodazole then allows microtubule ends to target FA and cause destabilisation, resulting in smaller area (Fig. S3). We show that in *Cyri-b* KO cells, FAs do not enlarge in the presence of nocodazole, nor does nocodazole washout have any detectable effect on their destabilisation (Fig. S3). Here, we conclude that a lack of microtubule plus ends tracking into the cell periphery could underly the reduced ERC1 localisation at the leading edge of cells and account for the reduced FA turnover we observed in CYRI-B knockout cells.

Overall, this suggests that the over-active actin cytoskeleton in the *Cyri-b* KO cells might inhibit access of microtubule ends to the FA, preventing removal of active β1-integrin by the ERC1–liprin-α1–Kank complex. Taken together with our previous study showing how CYRI proteins function in integrin internalisation via macropinocytosis (Le et al., 2021; Nikolaou et al., 2024), we hypothesise that actin dynamics and contractile function control access of microtubule ends to the leading edge of the cell. Microtubule access promotes the loosening up of FAs by ERC1–liprin-α1, which allows integrin internalisation and normal recycling function (Fig. S4). Thus, the actin and microtubule cytoskeleton linkage are crucial for coupling of integrin trafficking with leading edge dynamics.

## DISCUSSION

Although CYRI proteins are known to regulate leading edge actin dynamics via Scar/WAVE complex and RAC1, very little is known about how they might crosstalk with nascent adhesions forming in lamellipodia. We previously found that depletion of CYRI proteins led to excess active β1-integrin displayed on the cell surface, due to a reduction in internalisation via macropinocytic uptake (Le et al., 2021). However, it was unclear whether or how inhibition of integrin internalisation by macropinocytosis affected adhesion dynamics. Here, we find that depletion of CYRI-B enhances the size and changes the composition of FAs, leading to enhanced maturation and a fibrillar elongated appearance. *Cyri-b* KO cells spread more rapidly than controls and show more rapid accumulation of proteins, such as zyxin, that are hallmarks of mature adhesions (Zaidel-Bar et al., 2003). Adhesion dynamics are likely affected by the ability of CYRI to modulate RAC1 activation, but we found that RAC1 hyperactivation does not fully account for the phenotype of *Cyri-b* KO cells. We acknowledge that spatial regulation of RAC1 might be important as well, but our results also point towards an intriguing role for CYRI proteins in control of the crosstalk between FAs and microtubules. This might simply be in loosening the actin network at the cell edge to allow access to microtubules.

To better understand the mechanisms for enhanced FA maturation in *Cyri-b* KO cells, we performed a Bio-ID screen to identify proteins in proximity to paxillin in FAs of control versus knockout cells. Paxillin has one of the greatest numbers of protein binding partners within a FA and is ideal to use as the base for understanding proximal protein changes in the adhesions (Chastney et al., 2020; Zaidel-Bar et al., 2007). We found multiple targets that were potentially enriched in proximity to paxillin in the FAs of *Cyri-b* KO cells, suggesting a potential role in mechanosensing, maturation and contractility. Hits included Shroom2 and Shroom4, which are implicated in contractility via RhoA activation (Simoes et al., 2014); pragmin, a pseudokinase that promotes RhoA activation via the small GTPase Rnd2 (Tanaka et al., 2006); tensin3, which is implicated in promoting oncogenesis and as a component of fibrillar adhesions (Atherton et al., 2021); and vinexin and PAK2, both of which are implicated in mechanotransduction and force production (Campbell et al., 2019; Kuroda et al., 2018) (Fig. S2D,E). We also found that ERC1, a protein implicated in internalisation of FA proteins (Astro et al., 2016) was enriched in the proximity of paxillin in control adhesions over the knockouts.

Microtubule targeting to adhesions was originally shown to relax adhesions by Kaverina et al. (1999) and is thought to deliver proteins such as ERC1, liprin-α1, and LL5α (also known as PHLDB1) and LL5β, which dock and displace adhesion proteins to allow internalisation (Astro et al., 2016; Hotta et al., 2010). Owing to its role in adhesion turnover, we followed up ERC1 and confirmed that it was indeed depleted from the leading edge of *Cyri-b* KO cells and expressed at an ∼50% reduced level in *Cyri-b* KO cells. Furthermore, depletion of ERC1 by siRNA showed a similar phenotype to *Cyri-b* KO cells, supporting the idea that loss of CYRI-B impacts of FA turnover at least in part via ERC1. It remains an open question as to how loss of CYRI-B restricts ERC1 access to the cell leading edge. We reasoned that the excess actin assembly around the leading edge of *Cyri-b* KO cells might restrict access to the leading edge by the microtubule ends that were delivering ERC1. The enlarged adhesion sizes could also lead to positive feedback enhancing actin stress fibres and further obstructing ERC1 from accessing adhesion sites. We noticed a striking lack of EB1-positive microtubule ends tracking towards the periphery of many *Cyri-b* KO cells, supporting this hypothesis. Furthermore, if we weakened the actin network or the contractile myosin network with low doses of latrunculin-A or blebbistatin, we could rescue the delivery of microtubule ends to the periphery of the cell and rescue the effect of CYRI-B depletion. It is also possible that the 50% reduction in ERC1 expression in Cyri-b KO cells contributed to the increased stability of FAs (Fig. 4B,C).

Although our data support the idea that CYRI-B loss promotes actin cytoskeletal changes that prevent microtubule- and ERC1-induced dynamic disassembly of FAs, we acknowledge that our study has limitations. First, we have not shown direct docking of ERC1 at FAs, but rather leading-edge localisation that is disrupted in CYRI-B knockouts. Second, we did not detect a direct interaction between CYRI-B and ERC1, suggesting that the effect of CYRI-B deletion on ERC1 is indirect and likely due to cytoskeletal changes. Third, it is possible that the excessive spreading of *Cyri-b* KO cells could also contribute to the reduction of microtubule targeting to the edges of cells as they are simply farther away. We think that the most likely explanation for the effects of CYRI-B loss on FA dynamics is the combined effect of lack of targeting of microtubule tips to the leading edge of cells where nascent adhesions are forming with the previously described role of macropinocytosis of integrins (Le et al., 2021). Direct observation of ERC1 and integrin co-trafficking in normal and *Cyri-b* KO cells would be needed to establish this mechanism, which awaits future studies.

Taken together, our results suggest that CYRI proteins enhance dynamic actin turnover at the leading edge of the cell to allow microtubule and ERC1 access to the leading edge to accelerate FA dynamics. Disruption of this turnover by depleting CYRI-B leads to enhanced stability and maturation of FAs, which feeds back positively to enhance stress fibres and recruitment of pro-contractility proteins to FAs (Fig. S4). It will be interesting to know whether ERC1-mediated integrin internalisation is linked to macropinocytosis or whether these represent two separate and possibly additive mechanisms for mediating integrin internalisation from the cell surface.

## MATERIALS AND METHODS

### Mammalian cell culture conditions

Mouse embryonic fibroblasts (MEFs; isolated in our lab) and mouse melanoma B16-F1 cells (gifted from Klemens Rottner, TU Braunschweig, Germany) were maintained in Dulbecco's modified Eagle's medium (DMEM; Gibco, 21969-035) supplemented with 10% FBS (Gibco, 10270-106) and 2 mM L-glutamine (Gibco) at 37°C and 5% CO_2_ (denoted as complete DMEM). MEF complete DMEM was supplemented with 1 mg ml^−1^ primocin (Invivogen, ant-pm-05). Cells were routinely tested for *Myocoplasma* contamination (MycoAlert; Lonza).

### Transfection of mammalian cell lines

*Cyri-b^fl/fl^* MEFs were transiently transfected by electroporation (Amaxa, Kit T, program T-020) with 5 μg DNA and plated overnight to recover. B16-F1 cells were plated on a six-well plate and grown to 70% confluency and later transfected with Lipofectamine 2000 following the manufacturer's guidelines with 2–5 μg DNA.

### Genetic knockouts

Inducible knockout of *Cyri-b^fl/fl^* MEFs were generated by addition of 1 μM 4-hydroxytamoxifen (OHT) in the growth medium, with cells being split on day 2 and used in an assay on day 4 as described in Fort et al. (2018).

### Generation of *Cyri-b* KO B16-F1 cells

*Cyri-b* KO B16-F1 mouse melanoma cells were generated using the Cas9-GFP system and cell sorting. Specific gRNAs against mouse *Cyri-b* (ex3: 5′-CACCGGGTGCAGTCGTGCCACTAGT-3′) were cloned into the sPs-U6-gRNA-Cas9 (BB)-2A-GFP vector (Addgene #48138; Ran et al., 2013). B16-F1 cells were transiently transfected with Cas9-GFP vectors and FACS sorted for GFP-positive cells 36 h after transfection. The empty sPs-U6-gRNA-Cas9 (BB)-2A-GFP vector was transiently transfected in B16-F WT cells as a control. Stable clones were isolated and tested for deletion of CYRI-B by western blotting.

### siRNA knockdowns

*Erc1* was genetically knocked down in B16-F1 WT cells using specific siRNA oligonucleotides targeting Rab6ip (Erc1) (Qiagen; 1027416). The cells were transfected using Lullaby transfection reagent (OZ Biosciences) according to the manufacturer's instructions with a pool of 10 nM of *Mus musculus* Rab6ip siRNA (2.5 nM each; Qiagen FlexiTube Gene Solution, 1027416) or a matched concentration of control scramble siRNA (AllStars Negative siRNA, Qiagen; 1027281). The knockdown efficiency of ERC1 was determined by western blotting using mouse anti-ELKS antibody (Sigma; E4531).

### SDS-PAGE and western blotting

Cell lysates were collected on ice by scraping cells in RIPA buffer [150 mM NaCl, 10 mM Tris-HCl pH 7.5, 1 mM EDTA, 1% Triton X-100, 0.1% SDS, 1× protease and phosphatase inhibitors (Thermo Scientific Halt™ Protease and Phosphatase Inhibitor Cocktail, 100×, 78446)]. The tubes were centrifuged for 10 min at 21,000 ***g*** and 4°C. The lysate was transferred to a clean Eppendorf tube and protein concentration was measured using Precision Red.

40 μg of protein lysate was resolved on NuPAGE Novex 4-12% Bis-Tris gels and transferred onto nitrocellulose membranes (Bio-Rad system). Membranes were blocked with 5% BSA in TBS-T (10 mM Tris-HCl pH 8.0, 150 mM NaCl and 0.5% Tween-20) for 20 min prior to overnight incubation with the primary antibody (see below) at 4°C on a shaking incubator. Membranes were then washed three times for 5 min each in TBS-T. Membranes were incubated at room temperature for 1 h with secondary DyLight-conjugated antibodies 680 and 800 (Thermo Fisher Scientific). The blots were washed again for 5 min in TBS-T three times before being imaged on the Li-Cor Odyssey CLx machine. Images were then analysed using the Image Studio Lite Version 5.2 and protein band intensities were calculated. These were then plotted in GraphPad Prism9 as a bar chart highlighting each repeat as a different shape and colour. A summary of all western blots used in our study is shown in Fig. S5.

### Immunofluorescence analysis

Cells were plated onto sterile 13 mm glass coverslips that had been previously coated with either 10 μg ml^−1^ rat tail collagen I (Corning, 356236; for MEFs) or 10 μg ml^−1^ laminin (Sigma, L2020; for B16-F1 cells). Cells were fixed with 4% PFA for 10 min at room temperature (RT). Coverslips were then washed three times with PBS before incubation with blocking buffer (0.05% Triton X-100, 5% BSA, PBS) for 15 min, with shaking. Primary and secondary antibodies (see below) were diluted in blocking buffer and incubated with the coverslips in a dark, humidified chamber for 1 h. Coverslips were washed three times in PBS and once in MilliQ water before mounting with FluoromountG solution containing DAPI (Southern Biotech; 0100-01).

### Antibodies and staining reagents

Full details of all antibodies and staining reagents used are given in Table S2.

### Microscopy imaging

Fluorescence images were acquired using a Zeiss 880 confocal microscope with Airyscan using a Plan-Apochromat 63×/1.4 oil DIC objective lens and 405 nm, 488 nm, 561 nm and 633 nm laser lines. Raw images were acquired and Airyscan processing was performed using Zen Black version 2.3 SP1. Alternatively, a Zeiss 710 confocal microscope using an EC Plan-NEOFLUAR 40×/1.3NA Oil DIC and 405 nm, 488 nm, 561 nm and 633 nm laser lines running on Zen Black version 2011 SP7 was used. Images were processed using Fiji Version 1.53q.

### Imaging and quantification of FAs

Cells were cultured as described above. The coverslips were fixed and stained with Alexa Fluor 647-conjugated phalloidin and mouse anti-Vinculin to measure cell area and FAs, respectively.

*Z*-stacked images were acquired using a Zeiss 880 confocal microscope with Airyscan using a Plan-Apochromat 63×/1.4 oil DIC objective lens and analysed using Fiji software. A maximum intensity projection (MIP) of the *Z*-stack image with 0.25 µm increments was performed, the FAs were enhanced using a Gaussian blur filter (2.0) and identified using ‘find maxima within tolerance’ in ImageJ. The output image from the ImageJ-derived maxima was overlaid onto a greyscale image of the FAs from the original file to indicate that the method can distinguish most FA proteins from the original image. Where erroneous structures were detected, manual deletion of the area was done before measurements. These were then measured using the ‘Analyse Particles’ plugin in Fiji to give FA area and length.

As an unbiased approach, we quantified morphological characteristics such as FA area using CellProfiler software (v2.4.0) applying the CellProfiler pipeline as described in Cutiongco et al. (2020), where FAs were identified by vinculin staining. The individual adhesions were measured for their area and displayed as a frequency graph using Orange 3.30.2 software.

### FA ratios

B16-F1 cells were grown on coverslips as described. The coverslips were fixed and stained with either mouse anti-vinculin or rabbit anti-vinculin antibodies. If the primary antibody used for focal adhesion markers was of mouse origin, rabbit anti-vinculin was applied to avoid species overlap. These antibodies served as a standard to define the focal adhesion zone, including markers such as rabbit anti-zyxin, mouse anti-talin-1, mouse anti-FAK, mouse anti-paxillin and rabbit anti-phospho-paxillin (Y31). Each FA was identified from the two antibody pairs – vinculin (as the standard) in combination with the other antibody of a different species. This created an FA area zone based on the sum of the two antibody pairings. Using the FIJI plugin Plot Profile tool, a line was drawn to measure the fluorescence intensity across the whole FA zone, starting from the lamellipodial tip region and extending back to the cytosol. The line profile included both antibody staining from lamellipodia to the cytosol, but the intensity of each antibody was calculated separately and graphed. A schematic of this is displayed in Fig. S1E. The fluorescence intensity of each antibody staining was first normalized, where the highest intensity reading for each antibody was given the 100% value and the subsequent values as a percentage of the highest. As all the FAs were of varying lengths, dividing the intensity reading into 100 equal parts normalized the plot profile for the length of the FAs. Therefore, antibodies with a fluorescence signal at the tip will have a high intensity peak at the start but have very little signal towards the rear of the FA. These were then plotted using GraphPad Prism to generate a heatmap. The graphical output provides an indication of the complexity of the FAs and where each protein is presented as the abundance from the periphery (tip) to cytosol (rear) of the FA. At least five FA measurements were taken across five or more cells for each antibody pairing.

### Active β1-integrin area

B16-F1 cells were plated on laminin-coated coverslips and left to spread. The coverslips were fixed and stained for rat anti-β1 integrin and Alexa Fluor 568-conjugated phalloidin. *Z*-stacked images with 0.25 µm increments were captured using a Zeiss 880 confocal microscope with Airyscan using a Plan-Apochromat 63×/1.4 oil DIC objective lens. In FIJI, a Gaussian filter was applied to the max projected images to reduce background and highlight the integrin signal. As there was a saturated signal in the cytoplasmic region around the nucleus that would affect the quantifications, we removed this region and focused the analysis on the lamella and lamellipodia regions of the cell. These were then measured using the ‘Analyse Particles’ plugin in FIJI to give β1 integrin area.

### Image-based active β1-integrin internalisation assay

This assay aims to quantify the internalisation of β1-integrin over time. B16-F1 cells were grown on laminin-coated coverslips overnight as described above. The next day, cells were washed once with ice-cold PBS and incubated with rat anti-β1-integrin antibody clone 9EG7 diluted in ice cold Hank's balanced salt solution (HBSS) for 1 h on ice in a dark humid chamber.

Active β1-integrin internalisation was induced by the addition of 1 ml of pre-warmed complete DMEM and quickly transferred to a 37°C incubator for specified times (10, 20 and 40 min). After the allotted time, the coverslips were washed once with ice-cold PBS and incubated for 5 min in stripping buffer (0.2 M acetic acid, 0.5 M NaCl, pH 2.5) to remove all extracellular bound antibody. The coverslips were washed a further time in ice-cold PBS and fixed with 4% PFA.

For the controls, a total active β1-integrin measurement was taken, whereby the cells were fixed prior to any antibody treatment. A second control to determine the efficiency of antibody stripping after incubation was the 0-min coverslip. Here, after incubation with the β1-integrin antibody, the coverslips were kept on ice, washed with the stripping buffer and not allowed to internalise. This control should not have any internalised β1-integrin.

After fixation, the coverslips were subjected to the immunofluorescence protocol as described above with only the blocking and permeablising step before the addition of the secondary antibody against rat IgG.

For the image acquisition, a *Z*-stack image was taken with a Zeiss 880 with AiryScan module using the Plan-Apochromat 63×/1.4 NA oil DIC objective lens. In FIJI, a maximum projection image was generated from the *Z*-stacked image with 0.16 µm increments, a Gaussian blur of 2.0 was applied to the image to reduce background noise. Manual thresholding was applied to the images and using the Analyse Particles plugin of FIJI to quantify the number of internalised active β1-integrin dots and the area of those dots normalised to the cell area. 40 fields of view were analysed from each condition over four independent experiments.

### CYRI-B–GFP-positive structures containing β1-integrin

B16-F1 cells were transiently transfected with CYRI-B–p17–GFP and mCherry–β1-integrin (Addgene #55064) and plated on laminin-coated glass bottom dishes. Images were acquired using a Zeiss 880 confocal microscope with Airyscan using a Plan-Apochromat 63×/1.4 NA oil DIC objective lens with a 37°C heated incubator, perfused with 5% CO_2_. Images were acquired every 10 s for 5 min. Images were processed using FIJI software and a 2.5 µm line through the structure was drawn and a plot profile intensity was captured. The intensities were then normalized where the brightest intensity was given a 100% value with the other values as a percentage of the highest value. Each structure was then averaged and displayed on a line graph using GraphPad Prism.

### FA formation and maturation

B16-F1 cells were trypsinised for 2 min and resuspended with complete DMEM and adjusted to 10^5^ cells per ml, with 500 µl added to each coverslip coated with laminin before being placed in the incubator for the specific times (10, 30 min, 1 and 3 h). The coverslips were gently fixed with 4% PFA to preserve the cells that had weakly attached. The coverslips were stained with mouse anti-paxillin as an early adhesion marker and rabbit anti-zyxin as a marker for more mature FAs and Alexa Fluor 647-conjugated phalloidin for cell area.

*Z*-stack images were acquired using a Zeiss880 microscope with AiryScan module, Plan-Apochromat 63×/1.4 NA oil DIC objective lens and 405 nm, 488 nm, 561 nm and 633 nm laser lines. The max intensity projection images from nine slices at 0.2 µm increments were analysed using Fiji and both paxillin and zyxin area and length was quantified over time to distinguish adhesion formation from nascent to mature FAs as described above. Data are presented from three independent experiments in SuperPlot format.

### FAs turnover

B16-F1 cells were transiently transfected with pEGFP-paxillin (Addgene #15233) as described above and plated onto 35 mm glass-bottom Ibidi dishes coated with laminin. Short movies of 1 frame per minute for 30 min were obtained using the 488 nm laser on the Zeiss LSM 880 confocal microscope with Airyscan module using a Plan-Apochromat 63×/1.4 NA oil DIC objective lens at 37°C and 5% CO_2_. Raw images were acquired and Airyscan processing was performed using Zen Black version 2.3 SP1. Time-lapse movies were processed using Fiji software 1.53q, where the image sequences were stabilized using the Fiji plugin Image stabilizer and a Gaussian blur 2.0 was applied to the image to highlight the FAs. If there were more than one cell imaged in a field of view, then this was edited to focus only on one cell throughout the duration of the movie. The movies were submitted to the focal adhesion analysis server (FAAS; https://faas.bme.unc.edu/; Berginski and Gomez, 2013) where a threshold of 2.5 units was maintained across all image sets and positive structures or 15 pixels^2^ that last for at least five consecutive frames were quantified as being a FA. Assembly and disassembly rates are presented as rates from the FAAS. FA lifetimes were also calculated using FAAS which equate to the birth and death of the adhesions. The datasets presented are the average assembly, disassembly or lifetime of all the adhesions within a single cell. Data presented from three independent experiments in SuperPlot format.

### xCELLigence cell spreading

E-plate 16 (Agilent; 5469830001) were coated with laminin overnight and equilibrated with DMEM complete for 30 min prior to imaging at 37°C. Cells were harvested and adjusted to 5×10^3^ per well. The cells were seeded in technical quadruplicate and the plate was immediately transferred to the Acea RTCA DP xCELLigence machine maintained at 37°C, 5% CO_2_. Cell index was measured at 5-min time intervals for 8 h and readings were averaged for each condition. The impedance between the electrodes and cells determined cell index over time. Cell index represents a measure of cell morphology, size and the strength of attachment to the surface of the plate. Quadruplicate readings were taken for each condition. Data are presented as the average impedance from three independent replicates as described in Whitelaw et al. (2020).

### BioID–paxillin

B16-F1 cells were stably transfected with GFP-BirA*-Paxillin (kindly gifted by Dr Ed Manser, Institute of Molecular and Cell Biology, Singapore) and a pPuro empty vector (gifted from Klemens Rottern, TU Braunschweig, Germany). The cells were first selected with puromycin (2 µg/ml) and then after cell survival, the cells were then FACS sorted for low to mid-range GFP expression. Cells were plated on 15 cm laminin-coated dishes and left to grow to ∼50% confluence overnight. The following day, the dishes were treated with either 50 µM biotin ligase or DMSO for another 16 h.

For purification of the biotinylated proteins, the dishes were washed twice with ice-cold PBS, with the cells being scraped off the dish in 300 µl lysis buffer (50 mM Tris-HCl pH 7.2, 1% NP-40, 0.1% SDS, 500 nM NaCl, 10 mM MgCl_2_, 5 mM EGTA, pH 7.5) and incubated in the tube for 10 min prior to centrifugation (20 min, 21,000 ***g***, 4°C). The protein was then transferred to a clean tube and quantified using PrecisionRed (Cytoskeleton; ADV02-A) at OD_600_.

For each condition, 1.5 mg of protein was made to a volume of 500 µl in lysis buffer and added to 500 µl Tris-HCl pH 7.4 for a total 1 ml volume. This was then added to 50 µl Pierce NeutrAvidin Agarose bead slurry (Thermo Fisher Scientific; 29200) that was pre-washed twice with 250 µl lysis buffer. The tubes were then incubated overnight at 4°C on a rotating block. The next day, the tubes were spun at 264 ***g***, 4°C for 1 min and resuspended in Wash buffer 1 (2% SDS in distilled water). The tubes were then rotated for 8 min at room temperature due to high SDS content in Wash buffer 1. The Wash buffer 1 step was repeated and after the spin, the beads were resuspended in 1 ml Wash buffer 2 (0.1% sodium deoxycholate, 1% NP-40, 1 mM EDTA, 500 mM NaCl and 50 mM HEPES pH 7.5). The mixture was rotated for 2 min, then spun at pH 7.5and resuspended with 1 ml Wash buffer 3 (0.5% sodium deoxycholate, 0.5% NP-40, 1 mM EDTA, 250 mM LiCl, 10 mM Tris-HCl pH 7.4). The tubes were rotated for a further 2 min and after the spin, resuspended with 1 ml Tris-HCl. This wash step was repeated with 1 ml Tris-HCl and the beads were spun down. As much of the liquid was removed as possible, for mass spectrometry (MS) analysis.

For initial proof of concept, 2× sample buffer was added to the beads after the wash steps and heated to 100°C for 10 min. This was then run for western blot analysis and blots were probed using anti-streptavidin-HPR (Thermo Fisher Scientific; N100).

### Sample preparation

Agarose beads were resuspended in a 2 M urea and 100 mM ammonium bicarbonate buffer and stored at −20°C. On-bead digestion was performed from the supernatants. biological replicates (*n*=7) were digested with Lys-C (Alpha Laboratories) and trypsin (Promega) on beads as previously described (Hubner et al., 2010).

### MS analysis

Peptides resulting from all trypsin digestions were separated by nanoscale C18 reverse-phase liquid chromatography using an EASY-nLC II 1200 (Thermo Scientific) coupled to an Orbitrap Fusion Lumos mass spectrometer (Thermo Fisher Scientific). Elution was carried out at a flow rate of 300 nl/min using a binary gradient, into a 50 cm fused silica emitter (New Objective) packed in-house with ReproSil-Pur C18-AQ, 1.9 μm resin (Dr Maisch GmbH), for a total run-time duration of 135 min. Packed emitter was kept at 50°C by means of a column oven (Sonation) integrated into the nanoelectrospray ion source (Thermo Fisher Scientific). Eluting peptides were electrosprayed into the mass spectrometer using a nanoelectrospray ion source. An Active Background Ion Reduction Device (ESI Source Solutions) was used to decrease air contaminants signal level. The Xcalibur software (Thermo Scientific) was used for data acquisition. A full scan over mass range of 350–1550 *m*/*z* was acquired at 60,000 resolution at 200 *m*/*z*. Higher energy collisional dissociation fragmentation was performed on the 15 most-intense ions, and peptide fragments generated were analysed in the Orbitrap at 15,000 resolution.

### MS data analysis

The MS Raw data were processed with MaxQuant software (Cox and Mann, 2008) version 1.6.3.3 and searched with Andromeda search engine (Cox et al., 2011), querying SwissProt (UniProt, 2019) *Mus musculus* (62094 entries). First and main searches were performed with precursor mass tolerances of 20 ppm and 4.5 ppm, respectively, and MS/MS tolerance of 20 ppm. The minimum peptide length was set to six amino acids and specificity for trypsin cleavage was required. Cysteine carbamidomethylation was set as a fixed modification, whereas methionine oxidation, phosphorylation on serine-threonine-tyrosine, and N-terminal acetylation were specified as variable modifications. The peptide, protein and site false discovery rate (FDR) was set to 1%. All MaxQuant outputs were analysed with Perseus software version 1.6.2.3 (Tyanova et al., 2016).

Protein abundance was measured using label-free quantification (LFQ) intensities reported in the ProteinGroups.txt file. Only proteins quantified in all replicates in at least one group, were measured according to the LFQ algorithm available in MaxQuant (Cox et al., 2014). Missing values were inputted separately for each column, and significantly enriched proteins were selected using a two-tailed unpaired permutation-based *t*-test with FDR 5% or *P*<0.05.

Network BioID screen interactors was generated from LFQ intensities using the Hawaii plot functionality in Perseus (Rudolph and Cox, 2019). Different s0 and FDR% parameters were used in the multi-volcano analysis to define Class A (higher confidence, s0=5, FDR=0.01%) and Class B (lower confidence, s0=5, FDR=0.05%) potential interactors. One of the crucial points is the huge number of comparisons done during the *t*-test. In our project we quantify >940 proteins, therefore we did >940 comparisons in the *t*-test. If we consider a simple cut off at *P*<0.05 this gives 47 proteins flagged as significant merely by chance. To correct that we use a ‘permutation FDR’ analysis run alongside the *t*-test. This analysis will randomly switch condition and replicates to generate distribution of ‘false results’ and use those to control the false positives. The hits that satisfy those requirements (FDR) are indicated in the table in Fig. S2. We also selected hits to highlight based on functional relevance to FAs.

### GFP-Trap

Transiently transfected B16-F1 cells expressing GFP or CYRI-B–p17–GFP were washed twice with PBS on ice and scraped with 400 μl of lysis buffer [25 mM Tris-HCl, pH7.5, 100 mM NaCl, 5 mM MgCl_2_, 0.5% NP-40, 1× protease and phosphatase inhibitors (as above)]. Lysates were kept on ice 30 min and thoroughly mixed every 10 min. Soluble proteins were collected after a 10 min centrifugation step at 21,000 ***g*** and protein concentration was measured using PrecisionRed (Cytoskeleton; ADV02). 1.5 mg of protein was mixed with 25 μl of pre-equilibrated GFP-Trap_A beads (ChromoTek) and incubated for 2 h at 4°C with gentle agitation. Beads were then washed three times with 500 μl of wash buffer [100 mM NaCl, 25 mM Tris-HCl pH 7.5, 5 mM MgCl_2_].

To test for ERC1 interaction, 2× sample buffer and 2× reducing agent was added to the beads after the wash steps and heated to 100°C for 10 min. This was then run for western blot analysis and blots were probed using anti-ERC1 (Sigma).

### ERC1 and liprin localisation

B16-F1 cells were plated onto coverslips as above, fixed and stained with either Rabbit anti-ERC1 or chicken anti-liprin α1 and Alexa Fluor 647-conjugated phalloidin. Images were acquired using a Zeiss 710 confocal microscope and EC Plan-NEOFLUAR 40×/1.3 NA Oil DIC objective lens. The images were processed using FIJI software and the cells were scored for either a membrane or a more diffuse localization and presented as a percentage. Membrane localization was deemed positive when there was a tight localisation around the leading edge of the cell. Diffuse signals had no distinct localization anywhere in the cell and presented similar to a non-specific staining. For the line graph, a 3 μm line and subsequent plot profile of fluorescence intensity from the cell edge into the cytosol was taken. The fluorescence signals were averaged and plotted to represent both control and *Cyri-b* KO cells with either a membrane or diffuse localization.

### Actin photoactivation – retrograde flow

Photoactivation of actin and retrograde flow analysis was conducted as described in Papalazarou et al. (2020). Briefly, B16-F1 cells were transiently transfected with LifeAct-TagRed and PA-GFP-Actin (Addgene #57121) as described above. Imaging was conducted on a Zeiss 880 confocal microscope using a Plan-Apochromat 63×/1.4 NA oil DIC objective lens. The PA-GFP–Actin and LifeAct-TagRed were monitored with 488 nm and 568 nm lasers, respectively. A single pulse with a 405 nm laser (pulse length *t*=0.5 s) obtained photoactivation of actin at the ROI. Acquisitions were taken every second for 60 frames with an initial 5 s to obtain baseline GFP intensity prior to activation. Data presented as the means from three independent experiments in a time decay graph.

### Stress fibre quantification

The B16-F1 cells were plated onto coverslips coated with laminin and incubated overnight at 37°C and 5% CO_2_. The coverslips were fixed and stained with Alexa Fluor 647-conjugated phalloidin as described above. *Z*-stacked images obtained from a Zeiss880 microscope with AiryScan module, Plan-Apochromat 63×/1.4 NA oil DIC objective lens and 405 nm and 633 nm laser lines for DAPI and phalloidin, respectively. Images were processed using the macro to maximum project the z-stack, highlight the stress fibres with a difference of Gaussians threshold and for ridge detection to identify and quantify stress fibres as described in Whitelaw et al. (2020). Data presented from three independent experiments.

### Microtubule ends

pGFP-EB1 (Addgene #17234) was transiently transfected into the B16-F1 control and *Cyri-b* KO cells and imaged live on a Zeiss 880 microscope with Airyscan with a Plan-Apochromat 63×/1.4 NA oil DIC objective lens with the 488 nm laser at 1 image per second for 120 s. Image analysis was conducted using Fiji software to threshold for the EB1 microtubule tips. This number was then divided by the cell area.

Tracking of the EB1-positive tips was done using Fiji plugin TrackMaxima (IJ2). With setting the threshold to 8.0 and blur to 4.0. EB1 was tracked throughout the movie where the EB1 was in focus for at least 10 frames.

To measure the area of the lamellipodia absent of microtubules, the above movies were time-projected using the Fiji TrackMaxima (IJ2) software. The whole-cell area in the field of view was thresholded and used as a mask. The time-projected EB1 tracks were used as a mask for how far the microtubules have travelled to the leading edge. The EB1 track mask was subtracted from the whole-cell area mask to obtain an area devoid of microtubules at the leading edge of the cell. This devoid area was normalised as a percentage of the total area of the cell.

### Chemical inhibitors

Low-dose latrunculin A (Merck; L5163) and blebbistatin (Sigma; B0560) treatments were used to disrupt the actin cytoskeleton and reduce cell contractility, respectively. Serial dilutions of the drugs or DMSO were added to B16-F1 *Cyri-b* KO cells to determine the concentration at which the cells were still able to form lamellipodia and show healthy morphological features. We established that treatment with either 50 nM LatA or 5 µM blebbistatin for 20 min prior to imaging was sufficient to rescue the phenotypes of the *Cyri-b* KO cells.

### Statistics and reproducibility

All datasets were analysed using GraphPad Prism version 9.3.1. Datasets were tested for normality and then analysed using the appropriate statistical test, as described in each figure legend. Where appropriate, SuperPlots were used (Lord et al., 2020). For this, each individual value was colour coded according to the experiment and the mean of each experiment were overlaid with larger symbols, also colour coded to experimental day. The statistical analysis was done on the experimental means and presented with s.e.m. Significance levels rejecting the null hypothesis are represented above figures where: NS, not significant (*P*>0.05), *P<0.05, **P<0.01, ***P<0.001 and ****P<0.0001. Where significance was not reached, nothing was added above the graphs.

## Supplementary Material

10.1242/joces.263646_sup1 Supplementary information

Table S1. Summary of Bio-ID screen using GFP-Bir-A-paxillin in control and CYRI-B (Fam49B) knockout cells.
Key: Fam49B-KO = cells with CYRI-B knockout; WT = Cells with wild-type CYRI-B; plus-Bio= biotin added to the reaction; minus-Bio = biotin not added to the reaction.
All results: Shows analysis by student's T-test for all combinations of the experimental setup.
WT(+)_Fam49KO(+)- Shows all statistics for comparison between WT and CYRI-B KO cells with added biotin.
WT(+)_Fam49KO(+) (Top 100)- Shows the top 100 hits for comparison between WT and CYRI-B KO cells with added biotin.
Chart - Shows a volcano plot of the statistical differences between the two conditons.
