# Supplementary material for: CYRI-B loss promotes enlarged mature focal adhesions and restricts microtubule and ERC1 access to the cell leading edge
Source: J Cell Sci. 2025 Nov 24;138(22):jcs263646. doi: 10.1242/jcs.263646 (PMC12718634; doi:10.1242/jcs.263646)
Supplement: Supplementary information [file joces-138-263646-s1.pdf]

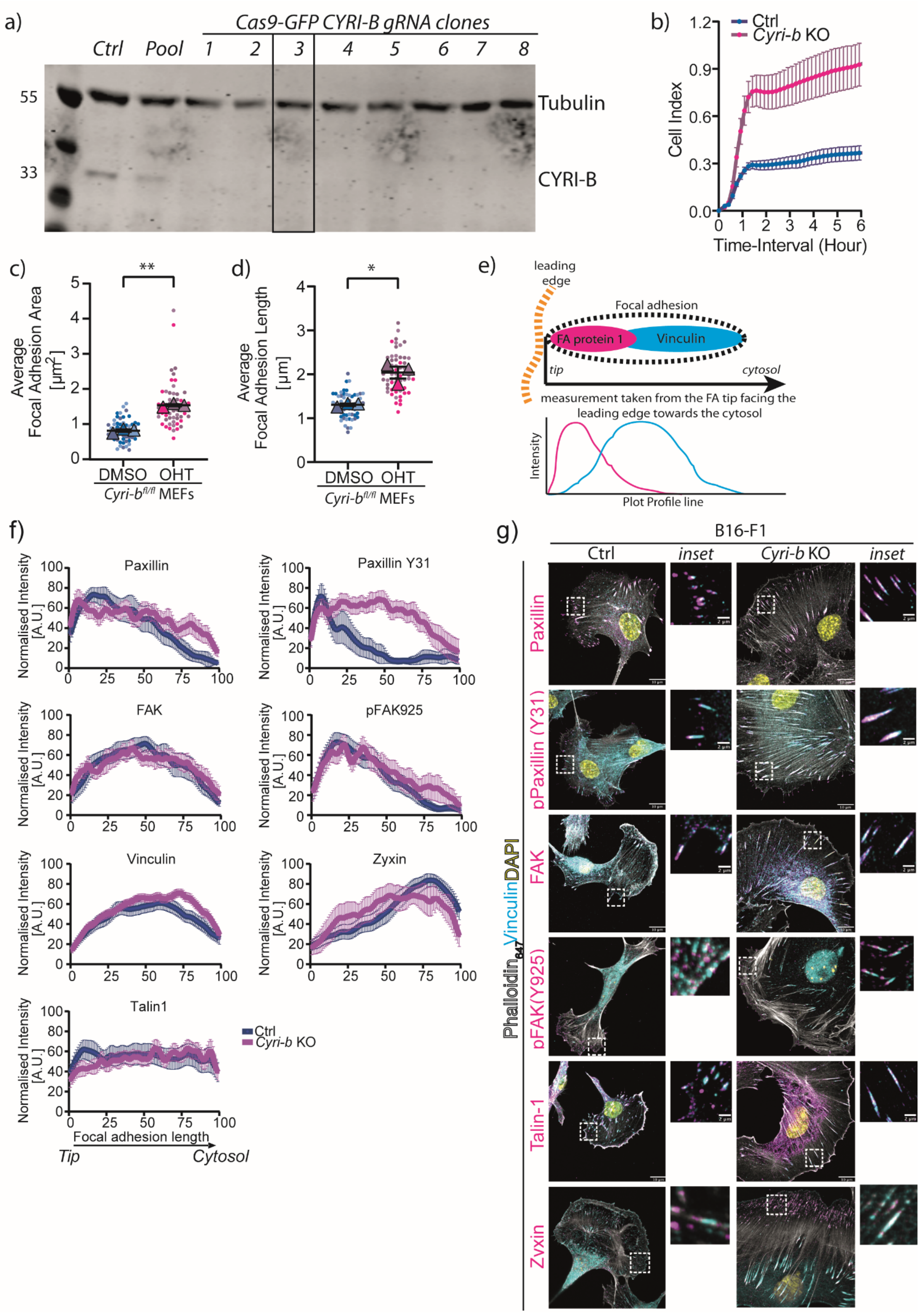

Journal of Cell Science • Supplementary information

Fig. S1.

a) Immunoblot of CRISPR-Cas9 knockouts of Cyri-b in B16-F1 cells. “Ctrl” denotes control lane for endogenous CYRI-B levels, “Pool” shows CYRI-B levels in the pool of GFP sorted cells after Cas9-treatment and the numbers refer to the clones isolated from the “pool” after sorting. For this study we used clone #3. Tubulin as loading control. **b)** Spreading of B16-F1 cells was investigated using an xCELLigence assay with readings of impedance (Cell Index, Y-axis) recorded every 10 minutes for 6 hours. Error bars represent Mean ± S.D. from 3 independent experiments with 4 technical replicates **c-d)** FA sizes in Cyri-$b^{fl/fl}$ MEFs ± 4-hydroxytamoxifen OHT as determined by vinculin staining. **c)** Average FA area and **d)** average FA length. 60 cells analysed for both DMSO and OHT treated MEFs from 3 independent experiments. Mean ± S.E.M., two-tailed paired t-test on the independent average from n=3 experiments in superplot format. * P<0.05, **P<0.01**. . e)** Schematic representations of two scenarios where the FA protein intensity was measured using ImageJ plot profile line tool. Left, where the comparative FA Marker is in-front of the vinculin marker. Right, where the comparative FA marker is towards to cytosol region of the vinculin marker. FAs were measured using the Fiji Plot Profile plugin from the tip (facing the leading edge) back to the rear of the FA (towards to cytosol). The intensity of each marker was measured independently along for the length of the whole FA (combined distance of both markers). **f)** Plot profiles of individual FA proteins where the intensity was normalised to the corresponding vinculin intensity. Line graphs depict the changes between the FA protein in control and Cyri-b KO cells. Error bars represent 95 % CI. g) Representative images with vinculin (cyan), the comparative FA antibody (magenta), actin cytoskeleton (white) and DAPI (yellow). Scale bars represent 10 µm and inset 2 µm.

Journal of Cell Science • Supplementary information

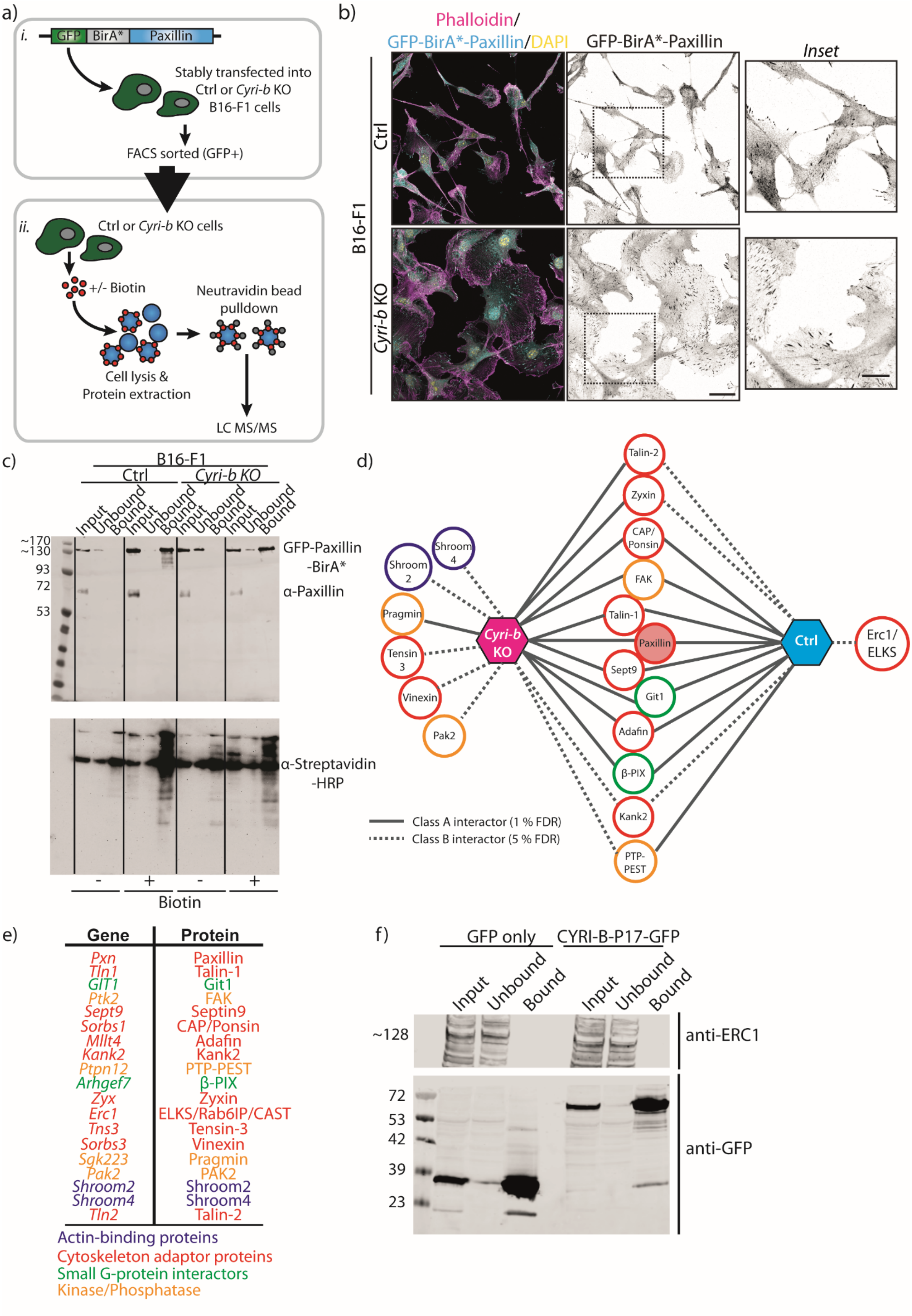

Journal of Cell Science • Supplementary information

Fig. S2.

a) Schematic diagram of BioID paxillin Mass spectrometry experiment. GFP-BirA*-Paxillin was stably transfected into B16-F1 cells and FACsorted for GFP expression. The cells were either spiked with or without biotin and the cells lysis was incubated with a Neutravidin bead slurry to pull-out biotinylated proteins. **b)** Localisation of stably expressed GFP-BirA*-Paxillin in B16-F1 cells. Scale bar represents 25 µm, inset scale bar 10 µm. **c)** Immunoblot of GFP-BirA*-Paxillin expression and the proteins biotinylated using anti-Streptavidin-HRP immunoblot staining, showing the input, the unbound and the bound fractions with or without biotin addition. **d)** Network of interactions from the BioID screen. Solid lines represent Class A interactors (1 % FDR) while dotted lines represent Class B interactors (5 % FDR). **e)** The gene symbol and protein name list of top interactors and their colour-coded functions.**)** **f)** GFP-trap pulldown of GFP only or CYRI-B-p17-GFP for their interactions with ERC1. Representative blot from n=2 experiments.

Journal of Cell Science • Supplementary information

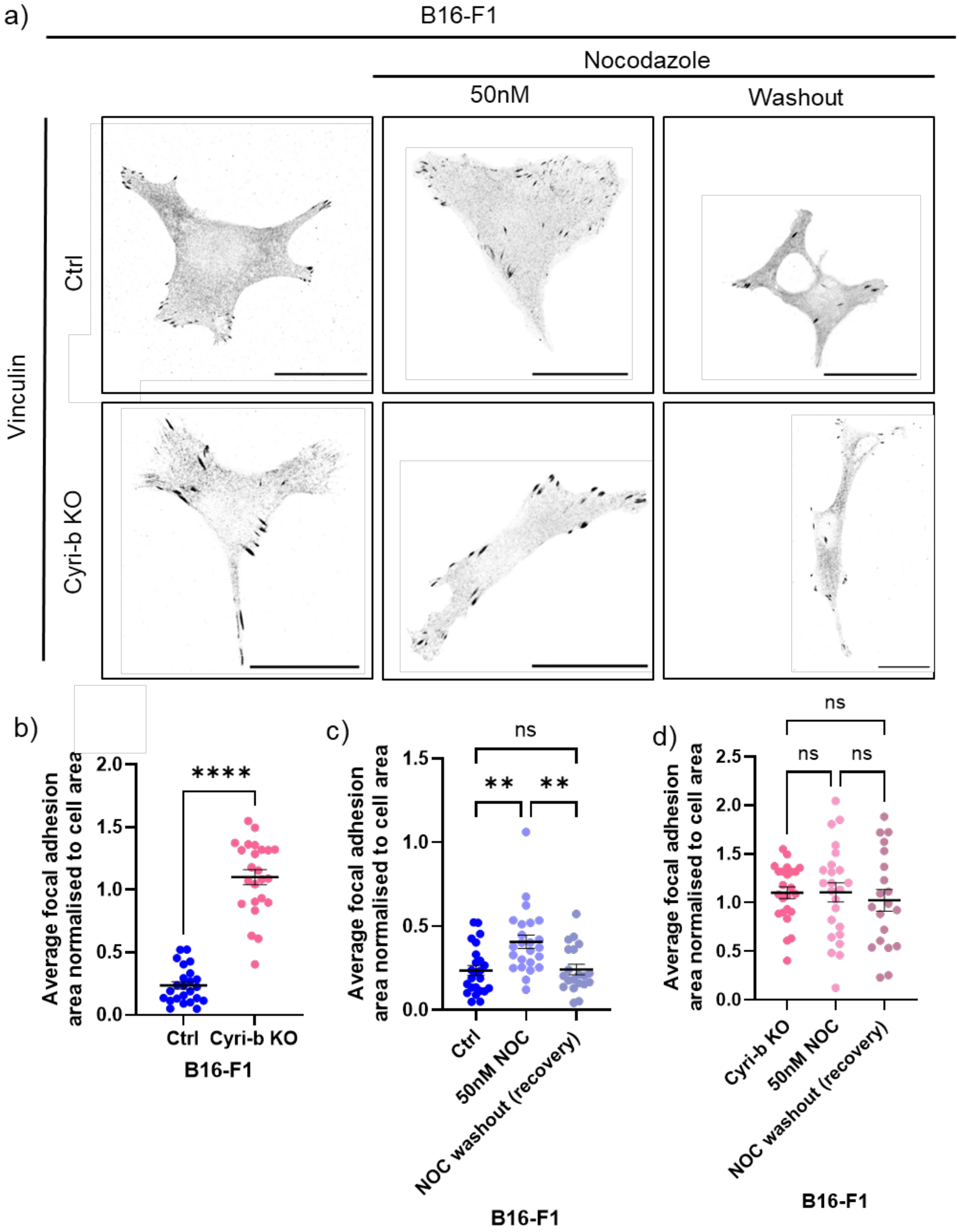

Journal of Cell Science • Supplementary information

Fig. S3. Loss of CYRI-B promotes stabilisation of focal adhesions preventing microtubule-based destabilisation

a) Representative images of cells stained with vinculin showing focal adhesion area in B16F1 control and Cyri-b KO cells treated with 50 nM nocodazole for 1 h. For recovery, cells were replaced with fresh media and imaged after 1 h. Scale bar 25 µm. **b)** FA area in B16F1 control and Cyri-b KO cells. **c)** FA area in B16F1 control treated with 50nM nocodazole and recovered. FA area in Cyri-b KO cells treated with 50nM nocodazole and recovered. A total of

24 cells in **d)** each condition and 20 cells from Nocodazole recovered were analysed from 3 independent experiments. Error bars represent Mean ± S.E.M. in superplot format. Statistical significance measured by a 1-way ANOVA, *P<0.05, **P<0.01, ***P<0.001, ****P<0.0001 and ns means no significance was reached.

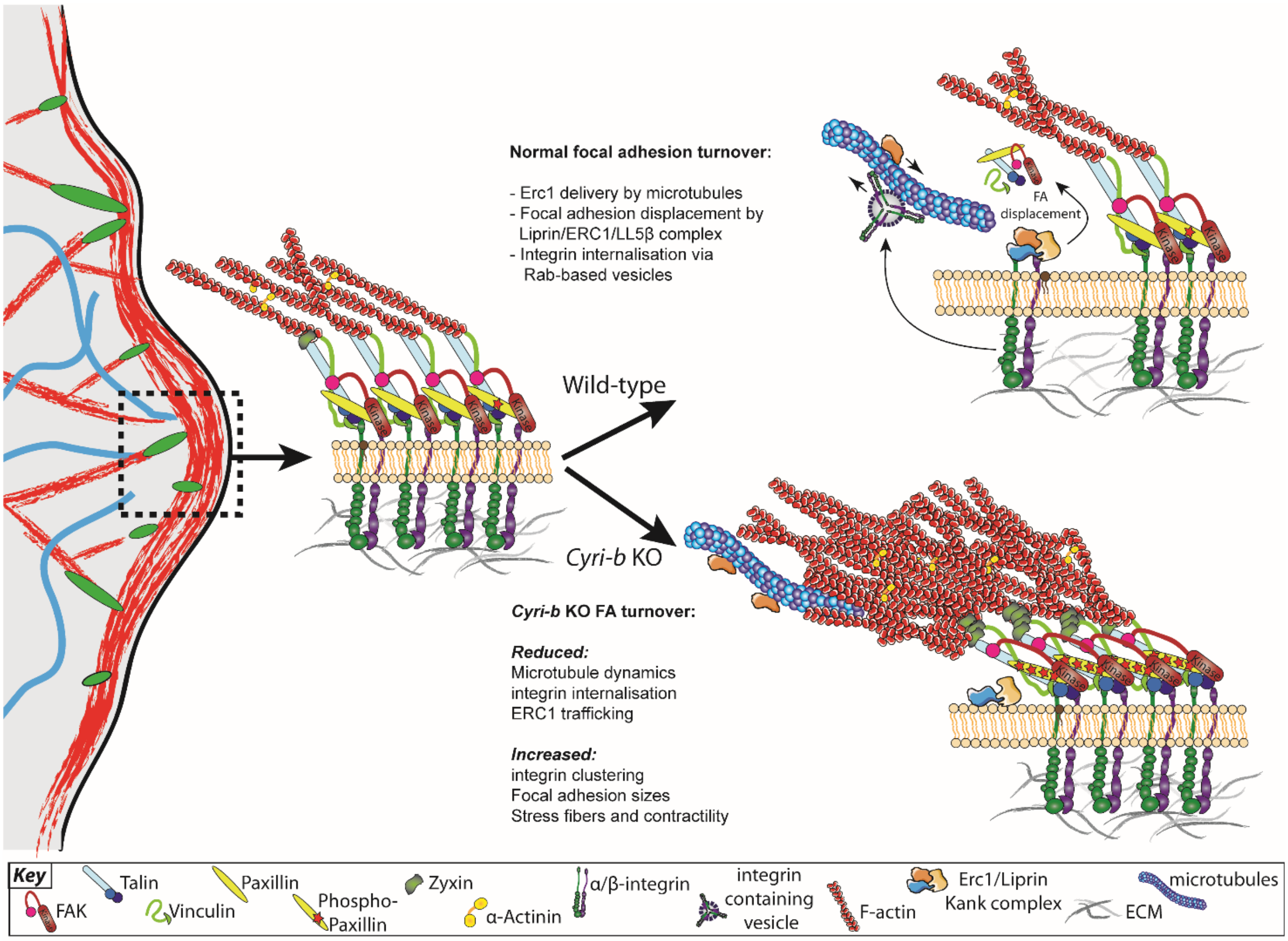

Fig. S4. Working model.

Engaged integrins at the leading edge form an initial focal complex, which matures to a FA to help transmit forces and pass signals to the cell. As the cell migrates, the FAs turn over. During this process, the ERC1/Liprin complex is delivered to the FA site by microtubules, where they in turn displace the intracellular part of the FA and allow the internalisation and recycling of the integrins. CYRI-B surrounds the internalised integrin vesicle and traffics back along the microtubules through macropinocytosis. In the Cyri-b KO cells; there is reduced microtubule dynamics and diffuse ERC1 localisation due to the mechanical constraints of the actin cytoskeleton. This in turn prevents the ERC1/Liprin complex from being docked at the FA site and displacing the FAs, subsequently causing more integrin clustering, increased FA sizes and actin stress fibers.

Figure 1a
CrispR *Cyri-b* KO

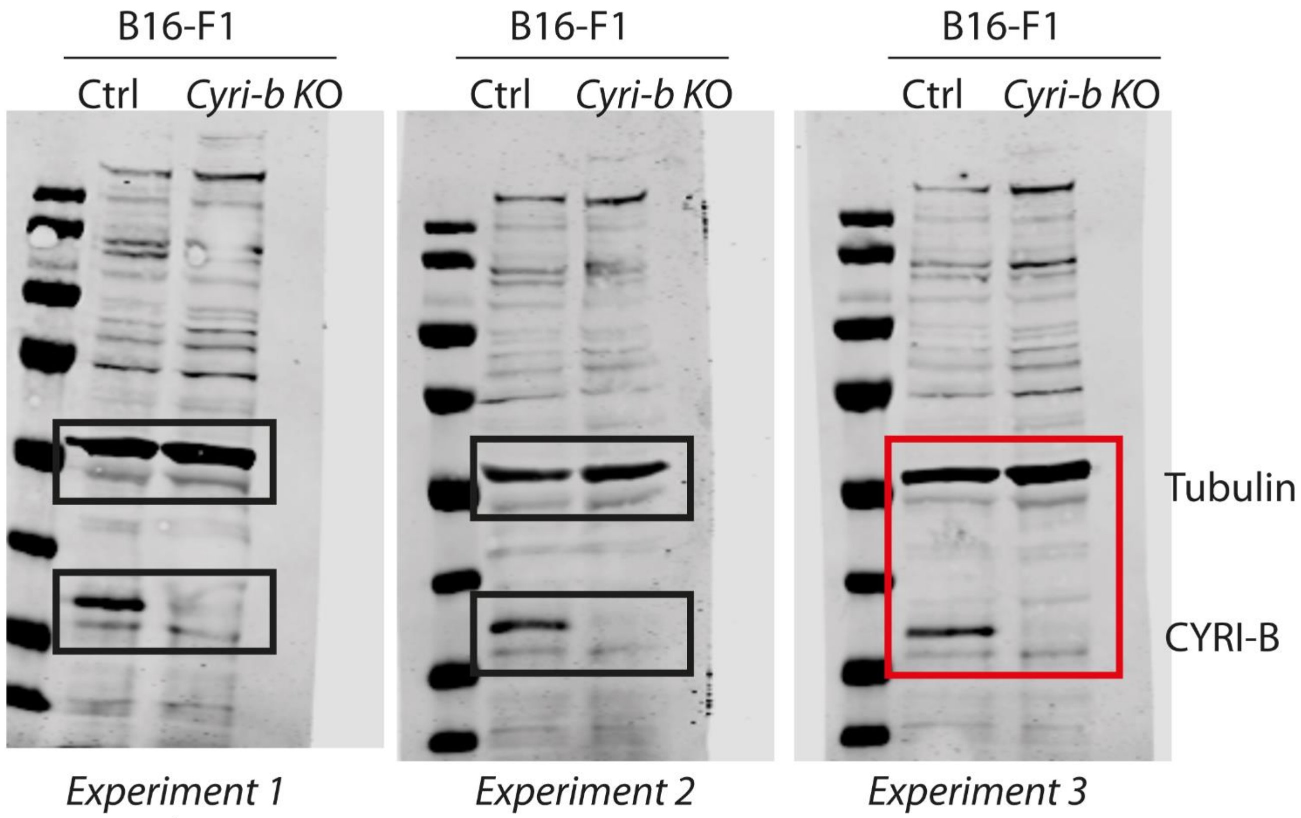

Figure 4b
ERC1 levels in B16-F1 Control and Cyri-b KO cells

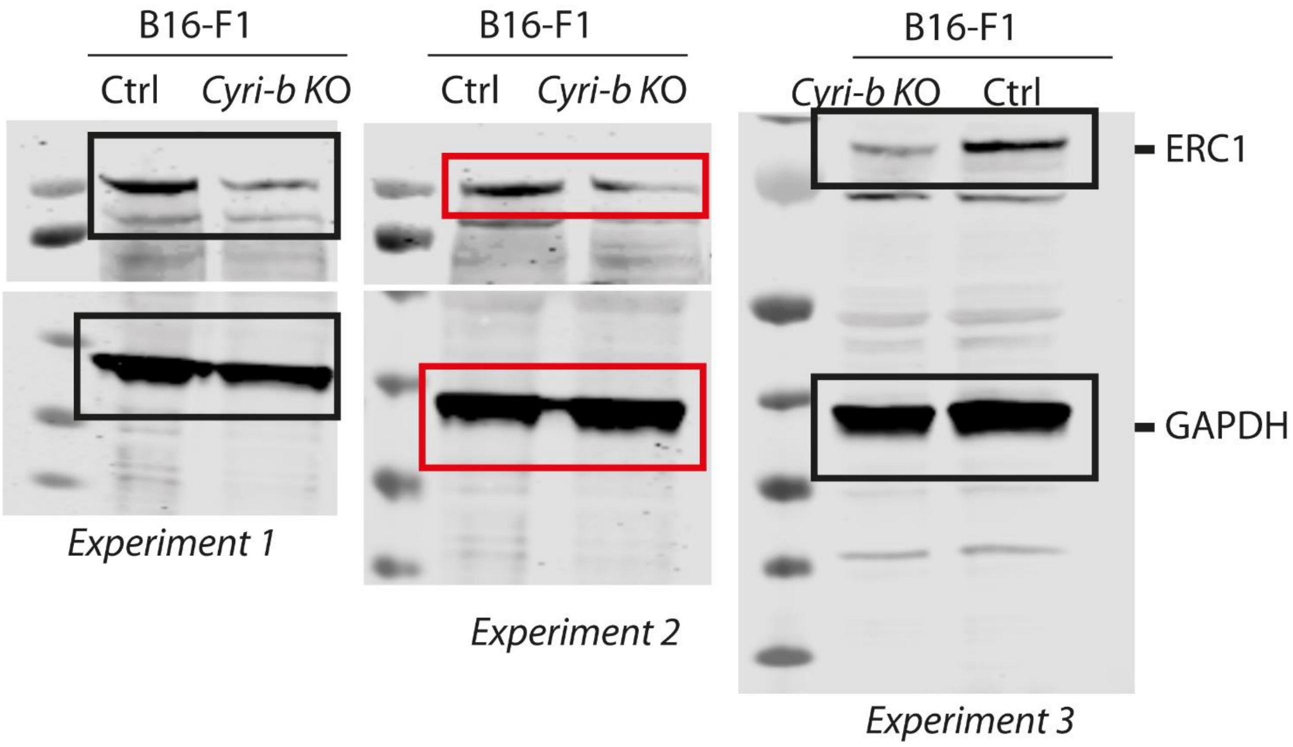

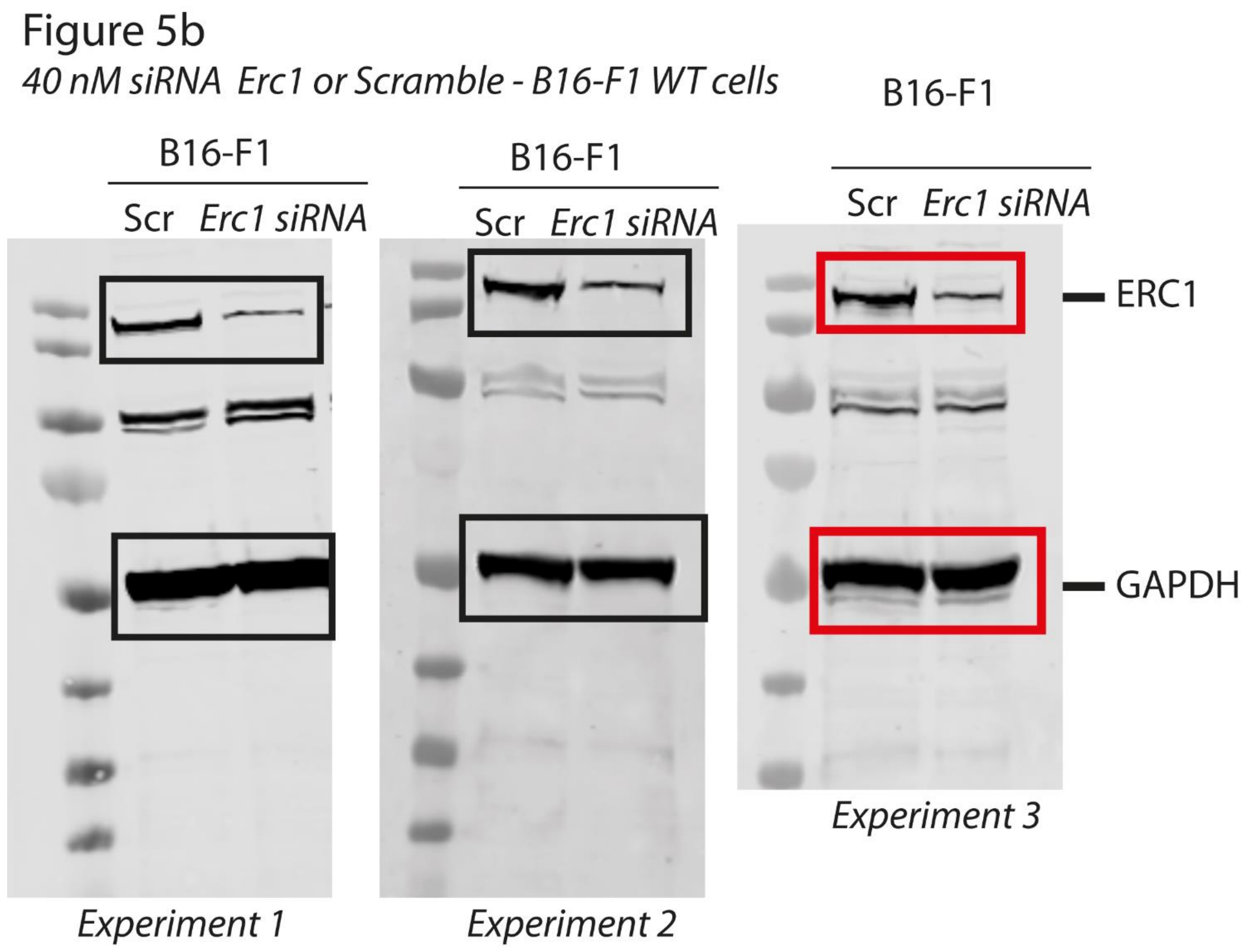
Figure 5b
40 nM siRNA Erc1 or Scramble - B16-F1 WT cells
B16-F1
Scr
Erc1 siRNA
B16-F1
Scr
Erc1 siRNA
B16-F1
Scr
Erc1 siRNA
ERC1
GAPDH
Experiment 1
Experiment 2
Experiment 3

Figure 6c

β1- integrin in B16-F1 Control and Cyri-b KO cells

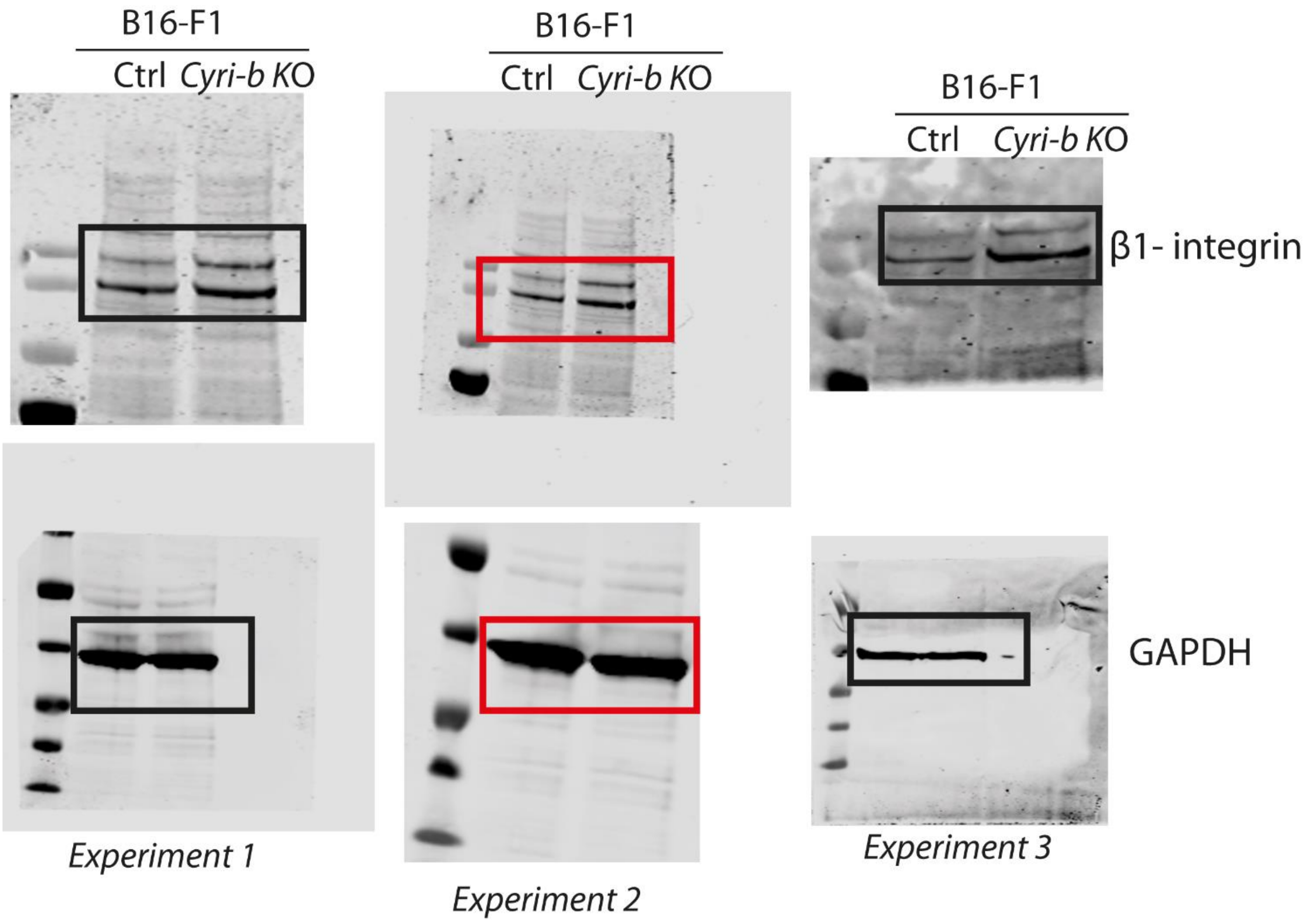

Journal of Cell Science • Supplementary information

Supplementary Figure 1a

B16-F1 Cyri-b KO tests

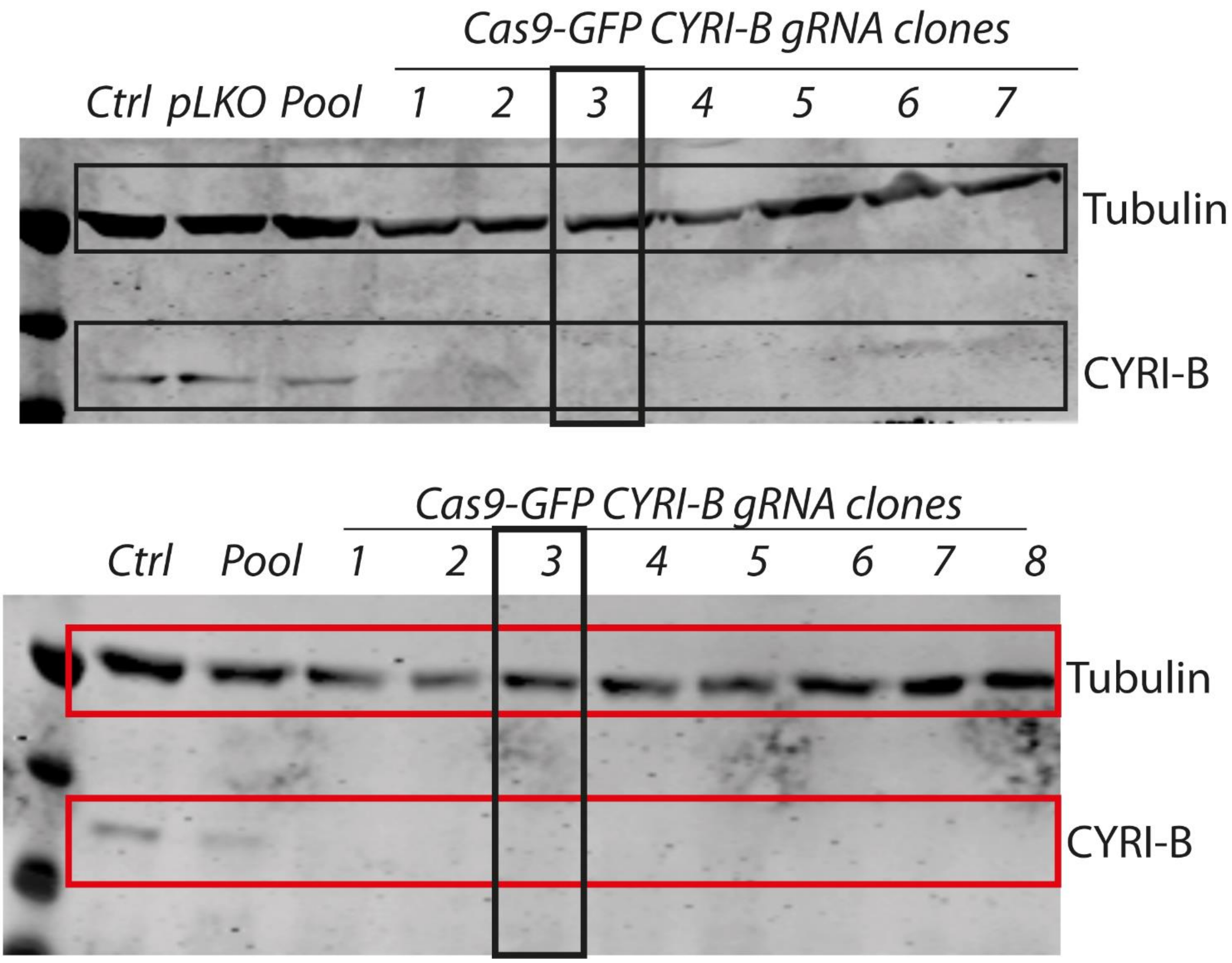

Supplementary Figure 2b

Strepavidin pulldown for biotinylated proteins
anti-Paxillin
anti-streptavidin

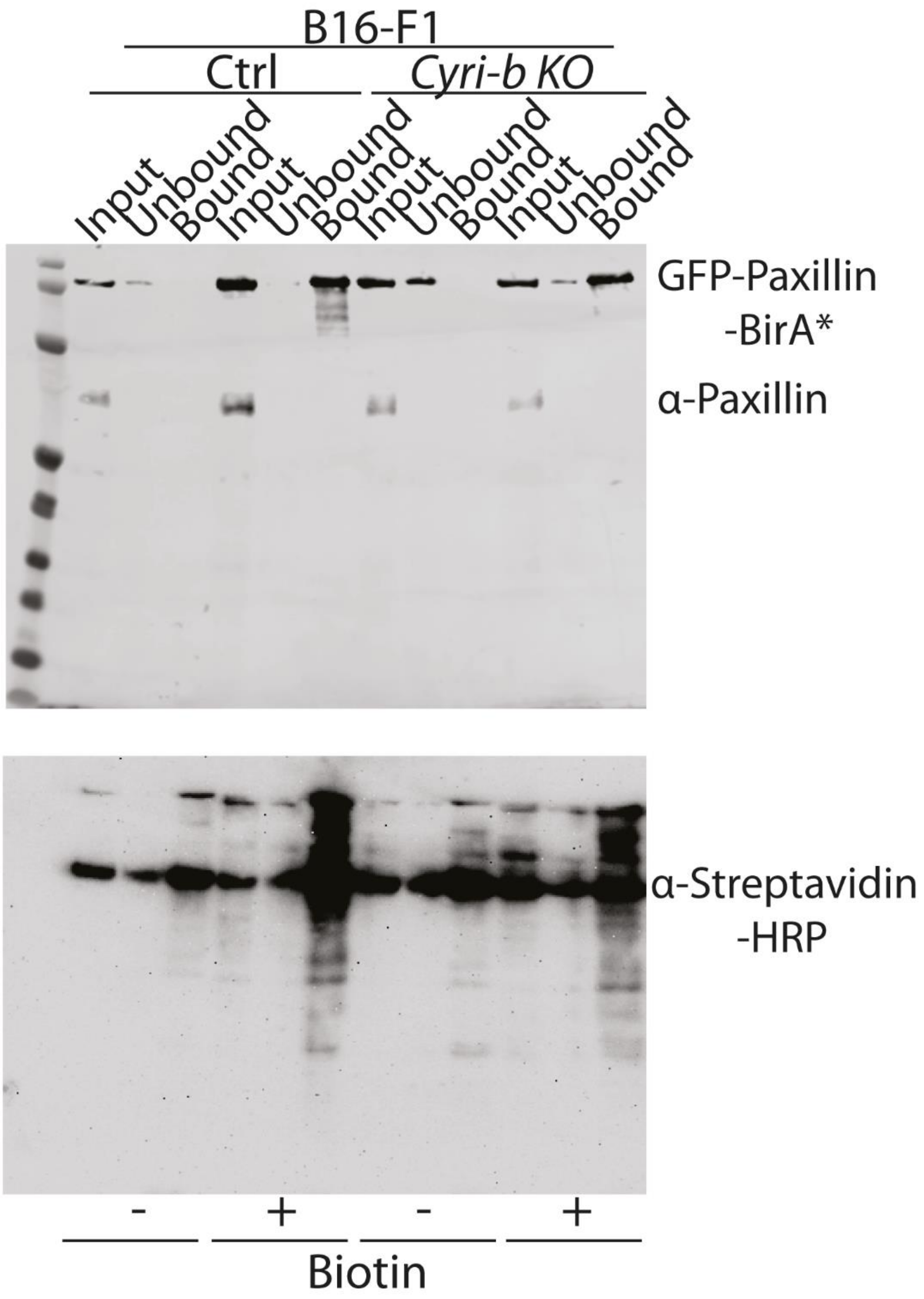

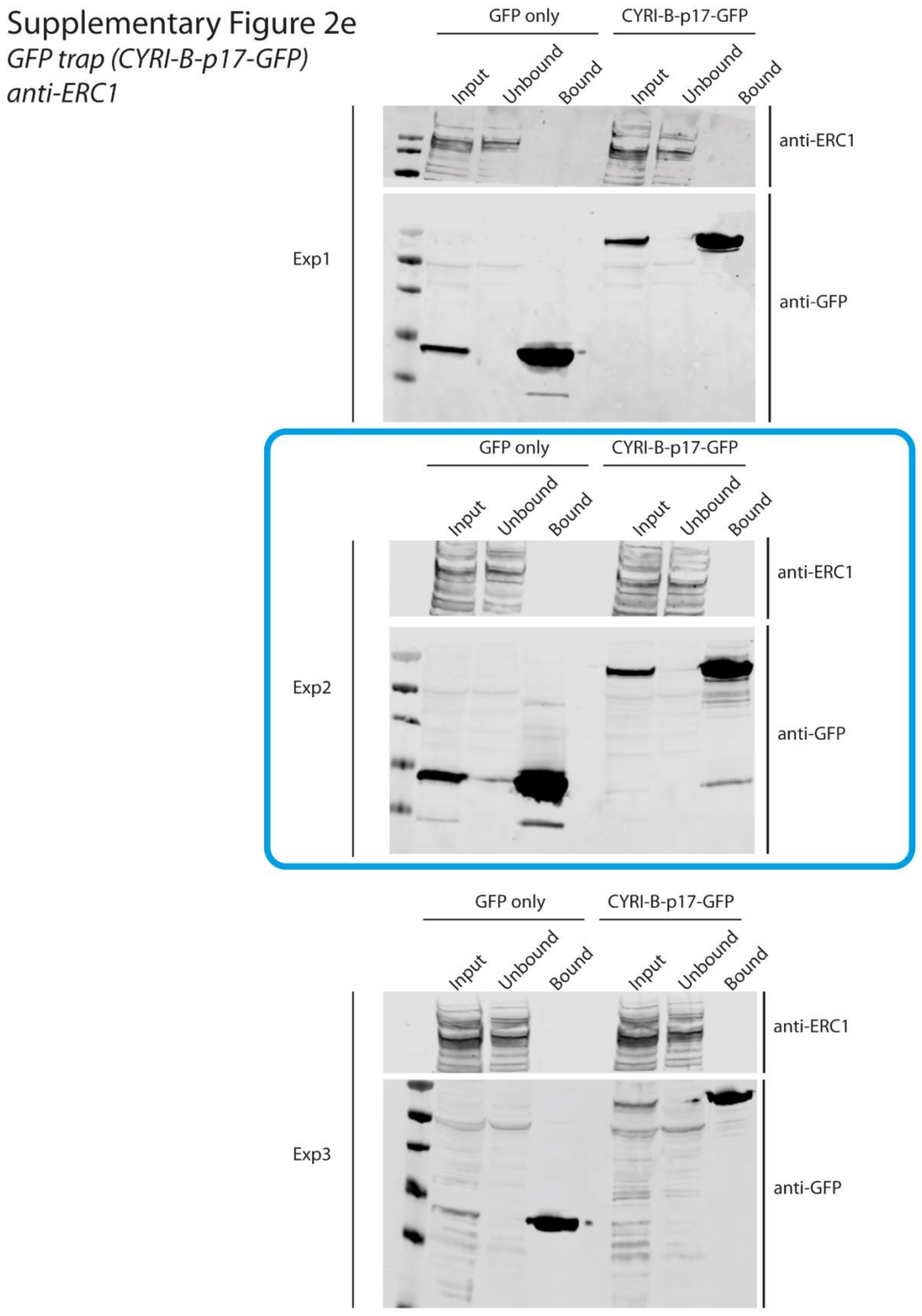

Fig. S5. Western Blots

Journal of Cell Science • Supplementary information

Table S1. Summary of Bio-ID screen using GFP-Bir-A-paxillin in control and CYRI-B (Fam49B) knockout cells.

Key: Fam49B-KO = cells with CYRI-B knockout; WT = Cells with wild-type CYRI-B; plus-Bio= biotin added to the reaction; minus-Bio = biotin not added to the reaction.

All results: Shows analysis by student's T-test for all combinations of the experimental setup.

WT(+)_Fam49KO(+)- Shows all statistics for comparison between WT and CYRI-B KO cells with added biotin.

WT(+)_Fam49KO(+) (Top 100)- Shows the top 100 hits for comparison between WT and CYRI-B KO cells with added biotin.

Chart – Shows a volcano plot of the statistical differences between the two conditons.

Available for download at

https://journals.biologists.com/jcs/article-lookup/doi/10.1242/jcs.263646#supplementary-data

Journal of Cell Science • Supplementary information

Table S2. Antibodies and Staining Reagents

Antibody / Reagent	Species	Manufacturer	Catalog No.	Dilution / Application
Vinculin (clone hVIN-1)	Mouse	Sigma	V9131	IF 1:400
Vinculin	Rabbit	Sigma	700062	IF: 1:200
Zyxin	Mouse	Abcam	ab50391	WB: 1:1000
Zyxin	Rabbit	Sigma	HPA004835	IF: 1:200
Talin1 (clone 8D4)	Mouse	Sigma	T3287	IF: 1:400
FAK	Mouse	Thermo Fisher Scientific	34Q36	IF: 1:400
Phospho-FAK (Y925)	Rabbit	Cell Signalling Technology	3284S	IF: 1:200
Paxillin	Mouse	BD Biosciences	610052	IF: 1:400
Phospho-Paxillin (Y31)	Rabbit	Thermo Fisher Scientific	44-720G	IF 1:200
β1-integrin	Rabbit	Cell Signalling Technology	4706	WB: 1:1000
β1 subunit of VLA	Rat	Millipore	1997	IF: 1:200
CD29 (clone 9EG7)	Rat	BD Pharmingen	553715	IF: 1:200
ELKS	Mouse	Sigma	E4531	WB: 1:1000
ERC1	Rabbit	Atlas Antibodies	HPA019523	IF: 1:200
PPFIA1/Liprin α1	Chicken	Abcam	ab26192	IF: 1:200
GFP	Chicken	Abcam	Ab13970	IF: 1:500
Fam49B	Rabbit	ProteinTech	20127-1-AP	WB: 1:1000
α-Tubulin (DM1A)	Mouse	Sigma	9026	WB:1:2000<br>IF: 1:500
GAPDH	Rabbit	Cell Signalling Technology	14C10	WB: 1:1000

Antibody / Reagent	Species	Manufacturer	Catalog No.	Dilution / Application
Alexa Fluor Conjugated Phalloidins	-	Thermo Fisher Scientific	AF488; A12379<br>AF568; A12380<br>AF plus 647; A30107	IF: 1:400

Secondary Antibody	Source	Cat. No.	Dilution
Goat anti-Mouse IgG (H+L) Cross-Adsorbed Secondary Antibody, Alexa Fluor™ 488	Thermo Fisher Scientific	A-11001	IF: 1:400
Donkey anti-Rabbit IgG (H+L) Highly Cross-Adsorbed Secondary Antibody, Alexa Fluor™ 488	Thermo Fisher Scientific	A-21206	IF: 1:400
Goat anti-Chicken IgY (H+L) Secondary Antibody, Alexa Fluor™ 488	Thermo Fisher Scientific	A-11039	IF: 1:400
Goat anti-Mouse IgG (H+L) Cross-Adsorbed Secondary Antibody, Alexa Fluor™ 568	Thermo Fisher Scientific	A-11004	IF: 1:400
Donkey anti-Rabbit IgG (H+L) Highly Cross-Adsorbed Secondary Antibody, Alexa Fluor™ 568	Thermo Fisher Scientific	A-10042	IF: 1:400
Goat anti-Rat IgG (H+L) Cross-Adsorbed Secondary Antibody, Alexa Fluor™ 568	Thermo Fisher Scientific	A-10077	IF: 1:400
Goat anti-Chicken IgY (H+L) Secondary Antibody, Alexa Fluor™ 568	Thermo Fisher Scientific	A-11041	IF: 1:400
Goat anti-Rabbit IgG (H+L) Cross-Adsorbed Secondary Antibody, DyLight™ 800	Thermo Fisher Scientific	SA5-10036	WB: 1:10,000
Goat anti-Mouse IgG (H+L) Cross-Adsorbed Secondary Antibody, DyLight™ 800	Thermo Fisher Scientific	SA5-10176	WB: 1:10,000
Donkey anti-Rabbit IgG (H+L) Cross-Adsorbed Secondary Antibody, DyLight™ 680	Invitrogen	SA5-10042	WB: 1:10,000
Donkey anti-Mouse IgG (H+L) Cross-Adsorbed Secondary Antibody, DyLight™ 680	Invitrogen	SA5-10042	WB: 1:10,000
Streptavidin-HPR	Thermo Fisher Scientific	N100	WB: 1:10,000

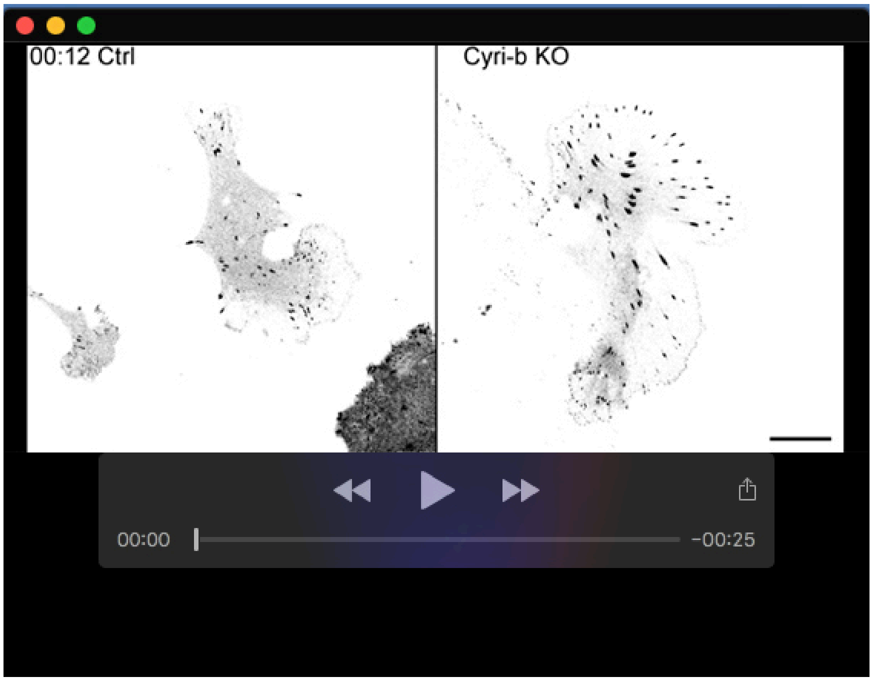

Movie 1. Focal adhesion turnover of control and *Cyri-b* KO cells.

Live imaging of B16-F1 control and Cyri-b KO cells expressing pEGFP-Paxillin. 1 image acquired every minute for 30 minutes to analyse focal adhesion dynamics. Playback rate at 5 frames per second. Scale bar 25 *μm.*

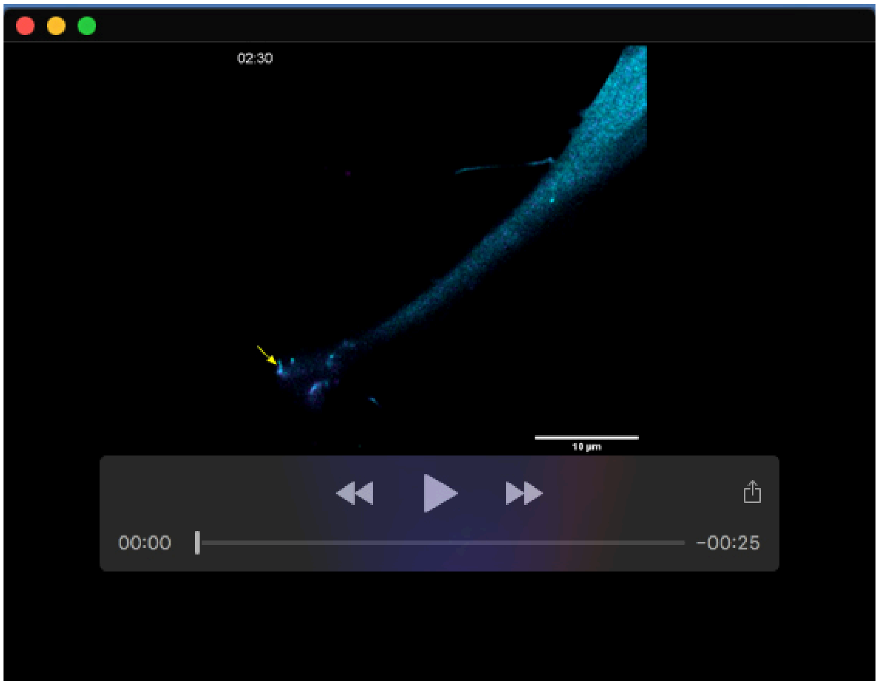

Movie 2. Integrin internalisation in B16-F1 *Cyri-b* KO cells rescued with CYRI-B p17-GFP.

Live imaging of B16-F1 Cyri-b KO cells rescued with CYRI-B-p17-GFP (Cyan) and β1-integrin-mCherry (Magenta). Yellow arrowheads highlight β1-integrin positive structures surrounded by CYRI-B. 1 image acquired every 10 seconds and playback at 7 frames per second. Scale bar represents 10 μm.

Journal of Cell Science • Supplementary information

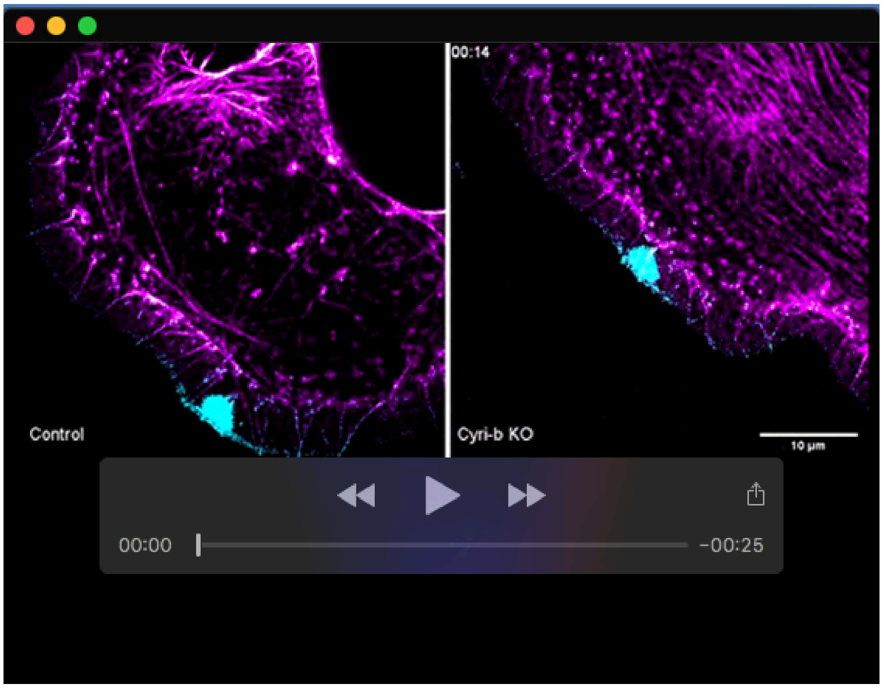

Movie 3. Actin retrograde flow in B16-F1 control and Cyri-b KO cells.

Photoactivation of PA-GFP-Actin (Cyan) and the actin cytoskeleton shown using LifeAct-TagRed (Magenta) in B16-F1 control (left) and Cyri-b KO (right) cells. 1 image acquired every 1 second and playback at 6 frames per second. There is an initial 5 second delay prior to photoactivation with the 405 nm laser to obtain baseline GFP intensity. Scale bar represents 10 µm.

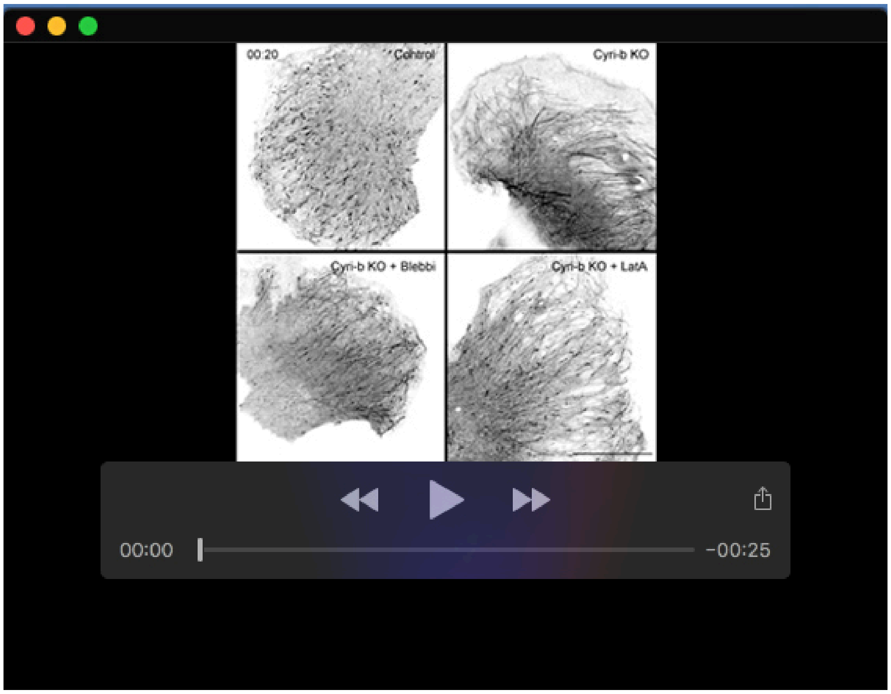

Movie 4. EB1 growth rates.

EB1 growth rates measured using pGFP-EB1 in B16-F1 control and Cyri-b KO cells with inhibitors (LatruculinA or Blebbistatin). 1 image per second for 120 seconds and playback at 10 frames per second. Scale bar represents 25 µm.

Journal of Cell Science • Supplementary information
